# Large Deflection Analysis of Peripherally Fixed Circular Membranes Subjected to Liquid Weight Loading: A Refined Design Theory of Membrane Deflection-Based Rain Gauges

**DOI:** 10.3390/ma14205992

**Published:** 2021-10-12

**Authors:** Jun-Yi Sun, Qi Zhang, Ji Wu, Xue Li, Xiao-Ting He

**Affiliations:** 1 School of Civil Engineering, Chongqing University, Chongqing 400045, China; 202016021045@cqu.edu.cn (Q.Z.); 202116131099t@cqu.edu.cn (J.W.); 20161602025t@cqu.edu.cn (X.L.); hexiaoting@cqu.edu.cn (X.-T.H.); 2 Key Laboratory of New Technology for Construction of Cities in Mountain Area (Chongqing University), Ministry of Education, Chongqing 400045, China

**Keywords:** circular membrane, liquid weight loading, fluid–structure interaction, integro-differential equation, power series method, closed-form solution

## Abstract

The anticipated use of elastic membranes for deflection-based rain gauges has provided an impetus for this paper to revisit the large deflection problem of a peripherally fixed circular membrane subjected to liquid weight loading, a statics problem when the fluid–structure interaction of membrane and liquid reaches static equilibrium. The closed-form solution of this statics problem of fluid–structure interaction is necessary for the design of such membrane deflection-based rain gauges, while the existing closed-form solution, due to the use of the small rotation angle assumption of the membrane, cannot meet the design requirements for computational accuracy. In this paper, the problem under consideration is reformulated by giving up the small rotation angle assumption, which gives rise to a new and somewhat intractable nonlinear integro-differential equation of the governing out-of-plane equilibrium. The power series method has played an irreplaceable role in analytically solving membrane equations involving both integral and differential operations, and a new and more refined closed-form solution without the small rotation angle assumption is finally presented. Numerical examples conducted show that the new and more refined closed-form solution presented has satisfactory convergence, and the effect of giving up the small rotation angle assumption is also investigated numerically. The application of the closed-form solution presented in designing such membrane deflection-based rain gauges is illustrated, and the reliability of the new and more refined closed-form solution presented was confirmed by conducting a confirmatory experiment.

## 1. Introduction

Membranes are increasingly being used in a wide variety of applications [1,2,3,4]. In our earlier work [5], the statics problem of fluid–structure interaction of a peripherally fixed circular membrane subjected to liquid weight loading is investigated analytically. The mathematical formulation of this problem results in a boundary value problem including both differential operation and integral operation, and the resulting integro-differential equations are successfully solved by using the power series method. Our primary motivation for investigating this fluid–structure interaction problem is to provide the closed-form solution needed for the development of a new type of membrane deflection-based rain gauge, a device for collecting and measuring the amount of rain which falls. In this study, the statics problem of fluid–structure interaction addressed in [5] is reformulated and solved, with an aim of giving a more refined closed-form solution than that given in [5], which is essential for the development of this membrane deflection-based rain gauge.

**Figure 1:**
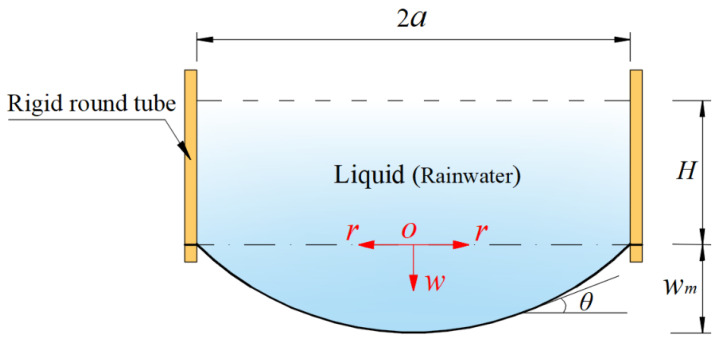
Geometry of the circular membrane under liquid weight loading.

Many membranes can exhibit large elastic deflection under transverse loading [6,7,8], which provides the possibility for the development of deflection measurement-based devices [9,10,11,12,13]. Figure 1 shows the circular rainwater storage container of the membrane deflection-based rain gauge to be developed, a vertically placed rigid round tube of finite length with an inner radius *a*, whose upper end is open and whose lower end is sealed by an initially flat, elastic circular membrane of radius *aˆ* which is used as an elastic bottom. The circular membrane as the elastic bottom will exhibit elastic deflection as the rainwater collected is injected into the storage container from the upper open end. Obviously, the higher the height *H* of the rainwater stored in the container, the greater the maximum deflection *w_m_* of the circular membrane, see Figure 1. If the circular membrane problem shown in Figure 1, i.e., the problem of axisymmetric deformation and deflection of the peripherally fixed circular membrane under liquid weight loading, can be analytically solved, then the analytical relationship between the maximum deflection *w_m_* and the volume (or the height *H*) of the rainwater in the storage container can be obtained. Therefore, with the obtained analytical relationship, the volume (or the height *H*) of the rainwater in the storage container can be determined by measuring the maximum deflection *w_m_*. The frequency (or time interval) of measuring the maximum deflection *w_m_* can be as small or as large as desired, that is, the maximum deflection *w_m_* can be measured once per second or minute or hour, providing convenience for different measurement data requirements. The rainfall per unit of time can be determined by two sets of measurement data, i.e., by the change in the volume (or the height *H*) of the rainwater in the storage containers divided by the time intervals of the two sets of measurement data. The rainfall per unit of time is calculated usually in minutes for rainstorms or heavy rain and usually in hours for moderate or light rain, but sometimes the volume of rainfall of moderate or light rain per minute also needs to be known. Therefore, it is best for rainfall to be measured in a continuous manner. However, traditional tipping bucket rain gauges (TBRGs) cannot achieve continuous measurement of rainfall, because the time it takes to fill a TBRG bucket with rainwater depends entirely on rainfall intensity [14,15]. Obviously, a TBRG bucket that can be filled with rainwater in a minute during light rain is not suitable for measuring the rainfall of rainstorm or heavy rain, because the volume of the bucket is too small and the tipping occurs too quickly during heavy rain, especially in a rainstorms. The advantage of the membrane deflection-based rain gauge proposed in this paper is that continuous measurements can be easily achieved (so, the rainfall per minute or hour can be easily given, regardless of whether one is measuring a rainstorm, heavy rain, moderate rain or light rain), but only if a closed-form solution can be given for the circular membrane problem in Figure 1.

Therefore, the key problem to be solved is to give the closed-form solution for the circular membrane problem shown in Figure 1. The large deflection phenomenon of membranes usually gives rise to nonlinear equations when formulated mathematically, and these nonlinear equations are generally difficult to address analytically [16,17,18,19,20,21]. In the existing literature, almost all analytical solutions for circular membrane problems are applicable only to the case of uniform loading, that is, loads applied onto the surface of circular membranes are always uniformly distributed regardless of membrane deflection [22,23,24,25,26,27,28,29]. However, shown in Figure 1, the loads (the liquid, rainwater) applied onto the surface of the circular membrane are not uniformly distributed. The nonuniformity of the liquid distributed on the circular membrane will vary with the height *H* of the liquid stored in the container. In other words, liquid applied onto the surface of the initially flat circular membrane gives rise to the deflection of the circular membrane, while in turn the shape of the deflected circular membrane determines the distribution of the liquid over the deflected circular membrane, i.e., the distribution of the loads acting on the deflected circular membrane. Therefore, there is an interaction between the action field of the fluid (rainwater) and the response field of the solid (membrane), resulting in the fluid–structure interaction or coupling phenomenon. Obviously, for a given quantity of liquid (i.e., the total volume of the rainwater injected into the storage container remains unchanged), the two-phase coupling interface will eventually reach static equilibrium, resulting in a statics problem of fluid–structure interaction.

This statics problem of fluid–structure interaction is analytically addressed in [5] for the first time, where the out-of-plane equilibrium equation, in-plane equilibrium equation, geometric equations and physical equations are established, and a closed-form solution of the problem is presented. The out-of-plane equilibrium equation is obtained by the equilibrium condition in the direction perpendicular to the initially flat circular membrane, while the in-plane equilibrium equation is obtained by the equilibrium condition in the direction parallel to the initially flat circular membrane. The geometric equations refer to the relationship between the strain and displacement of the deflected circular membrane, while the physical equations refer to the physical relationship between stress and strain following the generalized Hooke’s law. In [5], the out-of-plane equilibrium equation established is an integro-differential equation involving both integral and differential operations, which makes it difficult to analytically solve the simultaneous equations of out-of-plane and in-plane equilibrium equations, geometric equations and physical equations. The power series method has played an irreplaceable role in overcoming the difficulty of analytically solving these simultaneous equations, and due to its successful use, the closed-form solution for these simultaneous equations is finally given. The research results in [5] show that the fluid–structure coupling effect is substantial when the height *H* of the liquid (rainwater) in the storage container is relatively small, but it slowly becomes weak as the height *H* increases.

However, it can be seen from [5] that the integro-differential equation governing the out-of-plane equilibrium (i.e., Equation (4) in [5]) is actually derived under the small rotation angle assumption of a membrane—the rotation angle of the deflected circular membrane, *θ* (see Figure 1), is assumed to be so small that sin*θ* can be approximated by tan*θ*. That is, there exists an approximation of sin*θ* = tan*θ* under the small rotation angle assumption of the membrane. For the problem under consideration, since tan*θ* is equal to −d*w*/d*r* (*r* is the radial coordinate and *w* is the deflection, as in Figure 1, sin*θ* can be written as sin*θ* = tan*θ* = −d*w*/d*r*, i.e., Equation (3) in [5]. As is known to all, the exact relationship between sin*θ* and tan*θ* should be sin*θ* = 1/(1 + 1/tan^2^*θ*)^1/2^. Obviously, if sin*θ* = 1/(1 + 1/tan^2^*θ*)^1/2^ = 1/[1 + 1/(−d*w*/d*r*)^2^]^1/2^ (rather than sin*θ* = tan*θ* = −d*w*/d*r*) is used to establish the out-of-plane equilibrium equation, then the resulting the integro-differential equation governing the out-of-plane equilibrium will become more complicated mathematically, which can be seen in subsequent derivations (see Equations (3) and (4) in this paper). The more complex out-of-plane equilibrium equation naturally makes it more difficult to simultaneously solve the out-of-plane and in-plane equilibrium equation, geometric equations and physical equations, which is why sin*θ* = tan*θ* is used instead of sin*θ* = 1/(1 + 1/tan^2^*θ*)^1/2^ in [5].

Obviously, the use of small rotation angle assumption of a membrane using sin*θ* = tan*θ* instead of sin*θ* = 1/(1 + 1/tan^2^*θ*)^1/2^, will inevitably lead to the loss of computational accuracy of the closed-form solution of the statics problem of fluid–structure interaction shown in Figure 1, especially when the rotation angle of the deflected circular membrane, *θ*, is relatively large, i.e., when the height *H* of the liquid injected into the storage container is relatively large. Therefore, the closed-form solution presented by [5] is suitable only for a case when the height *H* of the liquid injected into the storage container is relatively small. In other words, if it is used when the height *H* of the liquid injected into the storage container is relatively large, a large calculation error will be caused. For a membrane deflection-based rain gauge to be developed, however, the height *H* needs to be able to range from a very small value to a very large value, which means that a closed-form solution whose computational accuracy is not affected by the change in the height *H*, is necessary. The closed-form solution presented by [5] cannot meet the requirement to develop such membrane deflection-based rain gauges, due to the use of small rotation angle assumption of the membrane, that is, using sin*θ* = tan*θ* instead of sin*θ* = 1/(1 + 1/tan^2^*θ*)^1/2^. Therefore, it is necessary to give up the small rotation angle assumption of the membrane, that is, using sin*θ* = 1/(1 + 1/tan^2^*θ*)^1/2^ (rather than sin*θ* = tan*θ*) during the derivation of the integro-differential equation governing the out-of-plane equilibrium. It can be seen from the following study that the closed-form solution which is obtained by giving up the small rotation angle assumption of the membrane does have the desired effect. The main aim of this study is to provide a closed-form solution without small rotation angle assumption, whose computational accuracy is not affected by change in the height *H*, in order to meet the requirement of developing such membrane deflection-based rain gauges.

In the following section, the fluid–structure interaction problem in Figure 1 is reformulated under the condition of using sin*θ* = 1/(1 + 1/tan^2^*θ*)^1/2^, resulting in a new and more complicated integro-differential equation governing the out-of-plane equilibrium. The problem reformulated is solved by using the power series method and a new, more refined closed-form solution of the problem is finally presented. In Section 3, some important issues are discussed, such as the validity and convergence of the closed-form solution presented. The variation of the difference between the closed-form solutions presented by [5] and by this paper with the increase of the height *H* is analyzed numerically. The application of the closed-form solution presented in designing such membrane deflection-based rain gauges is illustrated. In addition, in order to verify the validity of the closed-form solution presented, a confirmatory experiment is conducted. Concluding remarks are given in Section 4.

## 2. Membrane Equation and Its Solution

The circular rainwater storage container of the membrane deflection-based rain gauge to be developed is as shown in Figure 1, where a rigid round tube of finite length with inner radius *a* is placed vertically, such that the upper end of the round tube is open and the lower end of the round tube is sealed by an initially flat, elastic circular membrane with Young’s modulus of elasticity *E*, Poisson’s ratio *ν* and thickness *h* to form a soft bottom with the ability of elastic deformation, the rainwater collected is injected into the storage container from the upper end, and the maximum elastic deflection of the circular membrane eventually reaches *w_m_* when the height of the rainwater stored in the container reaches *H*.

**Figure 2:**
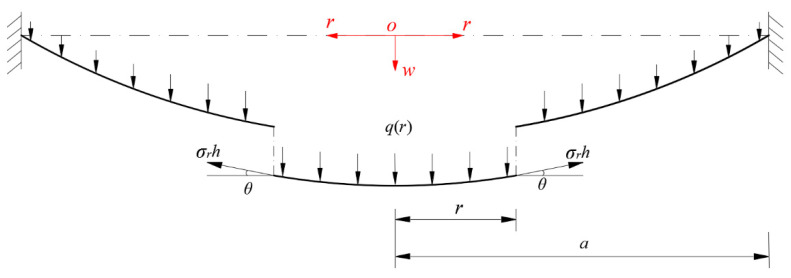
Sketch of a free body with radius 0 ≤ *r* ≤ *a*. Adapted from Refs. [18,29].

A piece of the central portion circular membrane whose radius is *0* ≤ *r* ≤ *a* is taken as a free body to study its static problem of equilibrium, as shown in Figure 2, where the origin *o* of the introduced cylindrical coordinate system (*r*, *φ*, *w*) is placed in the centroid of the geometric intermediate plane of the initially flat circular membrane, the polar coordinate plane (*r*, *φ*) is placed in the plane in which the geometric middle plane is located, *r* denotes the radial coordinate, *φ* denotes the circumferential angle coordinate which is not represented in Figure 2 due to the axisymmetry of the problem under consideration, *w* denotes the axial coordinate as well as the transverse displacement of the deflected circular membrane, *θ* denotes the rotation angle of the deflected circular membrane, *σ_r_* denotes the radial stress, and *q*(*r*) denotes the transverse loads that varies continuously with the radial coordinate *r* (i.e., the liquid acting on the surface of the deflected circular membrane, which is distributed uniformly in the circumferential direction and unevenly in the radial direction and can thus be represented as a function of the *r*).

The free body shown in Figure 2 is subjected to the joint actions of the external action force *F*(*r*) produced by the transverse loads *q*(*r*) within radius *r* and the total force 2*πrσ_r_h* produced by the membrane force *σ_r_h* acting on the boundary *r*. Obviously, the external force *F*(*r*) produced by *q*(*r*) within *r* is equal to the weight of the liquid within *r*, and is given by

(1) $$F(r)=\rho g\int_{0}^{r}[w(r)+H]\cdot 2\pi rdr=2\pi \rho g\int_{0}^{r}w(r)rdr+\rho g\pi r^{2}H,$$

where *w*(*r*) is the transverse displacement of the deflected circular membrane at *r*, *ρ* is the liquid density and *g* is the acceleration of gravity. The direction of *F*(*r*) is always vertically downward, that is, is always perpendicular to the initially flat circular membrane, while the vertical upward force is equal to 2*πrσ_r_h*sin*θ*, that is the vertical component of the force 2*πrσ_r_h* at *r*. Therefore, after ignoring the weight of the circular membrane, the equilibrium condition where the resultant force in the vertical direction is equal to zero gives

(2) $$2\pi r\sigma_{r}h\sin\theta =F(r)=2\pi \rho g\int_{0}^{r}w(r)rdr+\rho g\pi r^{2}H,$$

where

(3) $$\sin\theta =1/\sqrt{1+1/\tan^{2}\theta }=1/\sqrt{1+1/{\left(-dw/dr\right)}^{2}}.$$

Substituting Equation (3) into Equation (2) yields

(4) $$\frac{2r\sigma_{r}h}{\sqrt{1+1/{\left(-dw/dr\right)}^{2}}}=2\rho g\int_{0}^{r}w(r)rdr+\rho gr^{2}H.$$

In [5], this expression, which corresponds to Equation (3) in this paper, is given by sin*θ* = tan*θ =* −d*w*/d*r*, i.e., Equation (3) in [5]. It can be seen by comparing Equation (3) in this paper with Equation (3) in [5] that the approximation of replacing sin*θ* = 1/(1 + 1/tan^2^*θ*)^1/2^ with sin*θ* = tan*θ* has been discarded in this paper. Equation (4) is the fluid–structure coupling equation at static equilibrium, which is usually known as the out-of-plane equilibrium equation. Obviously, this integro-differential equation governing the out-of-plane equilibrium is much more complicated than the one presented in [5] (i.e., Equation (4) in [5]).

The in-plane equilibrium equation can be established by the equilibrium condition of the resultant force in the horizontal direction being equal to zero, and may be written as

(5) $$\frac{d}{dr}(r\sigma_{r}h)-\sigma_{t}h=0,$$

where *σ_t_* denotes the circumferential stress and *σ_t_h* is the circumferential membrane force.

Suppose that the radial strain is denoted by *e_r_*, the circumferential strain is denoted by *e_t_* and the radial displacement is denoted by *u*. Then, the geometric equations, the relations of strain and displacement, may be written as [22,23]

(6) $$e_{r}=\frac{du}{dr}+\frac{1}{2}{\left(\frac{dw}{dr}\right)}^{2}$$

and

(7) $$e_{t}=\frac{u}{r}.$$

In addition, the membrane is still assumed to be a linearly elastic or Hooke-type material. Thus the physical equations (i.e., the relations of stress and strain) follow the generalized Hooke’s law

(8) $$\sigma_{r}=\frac{E}{1-\nu^{2}}(e_{r}+\nu e_{t})$$

and

(9) $$\sigma_{t}=\frac{E}{1-\nu^{2}}(e_{t}+\nu e_{r}).$$

Eliminating *e_r_* and *e_t_* in Equations (8) and (9) by substituting Equations (6) and (7) into Equations (8) and (9) yields

(10) $$\sigma_{r}=\frac{E}{1-\nu^{2}}[\frac{du}{dr}+\frac{1}{2}{\left(\frac{dw}{dr}\right)}^{2}+\nu \frac{u}{r}],$$

and

(11) $$\sigma_{t}=\frac{E}{1-\nu^{2}}[\frac{u}{r}+\nu \frac{du}{dr}+\nu \frac{1}{2}{\left(\frac{dw}{dr}\right)}^{2}].$$

Eliminating d*u*/d*r* + (d*w*/d*r*)^2^/2 from Equations (10) and (11) and then eliminating *σ_t_* using Equation (5) yields

(12) $$\frac{u}{r}=\frac{1}{Eh}(\sigma_{t}h-\nu \sigma_{r}h)=\frac{1}{Eh}[\frac{d}{dr}(r\sigma_{r}h)-\nu \sigma_{r}h].$$

The usually consistency equation can be finally written by eliminating *u* from Equations (10) and (12), as

(13) $$r\frac{d}{dr}[\frac{1}{r}\frac{d}{dr}(r^{2}\sigma_{r}h)]+\frac{Eh}{2}{\left(\frac{dw}{dr}\right)}^{2}=0.$$

The specific solutions of the radial stress *σ_r_* and deflection *w* can be obtained from Equations (4) and (13), where the boundary condition, under which Equations (4) and (13) can be solved, are

(14) $$\frac{dw}{dr}=0\;\mathrm{at}\;r=0,$$

(15) $$\frac{u}{r}=\frac{1}{Eh}[\frac{d}{dr}(r\sigma_{r}h)-\nu \sigma_{r}h]=0\;\mathrm{at}\;r=a$$

and

(16) w=0 at r=a.

Let us proceed to the following nondimensionalization

(17) $$W=\frac{w}{a},\;S_{r}=\frac{\sigma_{r}}{E},\;S_{t}=\frac{\sigma_{t}}{E},\;x=\frac{r}{a},\;H_{0}=\frac{H}{a},\;G=\frac{\rho ga^{2}}{Eh},$$

and transform Equations (4), (5), (13)–(16), respectively, into

(18) $$4x^{2}S_{r}^{2}{\left(-\frac{dW}{dx}\right)}^{2}-G^{2}[{\left(-\frac{dW}{dx}\right)}^{2}+1]{\left[\int_{0}^{x}2xW(x)dx+x^{2}H_{0}\right]}^{2}=0,$$

(19) $$x^{2}\frac{d^{2}S_{r}}{dx^{2}}+3x\frac{dS_{r}}{dx}+\frac{1}{2}{\left(\frac{dW}{dx}\right)}^{2}=0,$$

(20) $$S_{t}\begin{array}{c} = \end{array}S_{r}+x\frac{dS_{r}}{dx},$$

(21) $$\frac{dW}{dx}=0\;\mathrm{at}\;x=0,$$

(22) $$\frac{u}{r}=(1-\nu )S_{r}+x\frac{dS_{r}}{dx}=0\;\mathrm{at}\;x=1$$

and

(23) W=0 at x=1.

*S_r_* and *W* can be expanded into the power series of the *x* due to the fact that the stress and deflection are both finite at *x* = 0, i.e., letting

(24) $$S_{r}=\sum_{i=0}^{\infty }c_{i}x^{i}$$

and

(25) $$W=\sum_{i=0}^{\infty }d_{i}x^{i}.$$

The recursion formulas for the coefficients *c_i_* and *d_i_* in Equations (24) and (25) can be determined by substituting Equations (24) and (25) into Equations (17) and (18), and the results in this way are that both *c_i_* and *d_i_* are always equal to zero when *i* is odd and can be represented as the polynomials of *c*_0_ and *d*_0_ when *i* is even, as in Appendix A and Appendix B.

The remaining two coefficients, *c*_0_ and *d*_0_, are usually known as the undetermined constants, and they can be determined by using the boundary conditions at *x* = 1 as follows. From Equation (24), the boundary condition Equation (22) gives

(26) $$(1-\nu )\sum_{i=0}^{\infty }c_{i}+\sum_{i=1}^{\infty }ic_{i}=0,$$

and from Equation (25), the boundary condition Equation (23) gives

(27) $$\sum_{i=0}^{\infty }d_{i}=0.$$

After substituting all the recursion formulas for the coefficients *c_i_* and *d_i_* into Equations (26) and (27) repeatedly, a system of equations containing only *c*_0_ and *d*_0_ can finally be obtained. As a result, the undetermined constants *c*_0_ and *d*_0_ can be determined by solving this system of equations, and with the known *c*_0_ and *d*_0_, the expressions of *S_r_* and *W* can also be determined. The problem under consideration is thus solved analytically.

## 3. Results and Discussions

The boundary condition, Equation (21), which has not been used yet, i.e., the condition of d*W*/d*x* = 0 at *x* = 0, can be used to confirm the validity of the above analytical process. The first derivative of the *W* versus the *x* can be obtained by the first derivative on both sides of Equation (25),

(28) $$\frac{dW}{dx}=\sum_{i=1}^{\infty }id_{i}x^{i-1}.$$

Equation (28) shows that d*W*/d*x* ≡ *d*_1_ when *x* = 0, while it can be seen from the derivation in Section 2 that *d*_1_ ≡ 0 because *d_i_* ≡ 0 when *i* is odd. Therefore, it may be concluded that d*W*/d*x* ≡ 0 at *x* = 0, which indicates that the boundary condition in Equation (21) can be naturally met by the closed-form solution obtained in Section 2. This to some extent indicates that the derivation in Section 2 is basically reliable.

### 3.1. The Convergence of the Power Series Solutions Obtained

Due to the complexity of the expressions of *c_i_* and *d_i_* (see Appendix A and Appendix B), the convergence of the power series solutions for radial stress and deflection obtained in Section 2 has to be discussed by examining the convergence of their specific solutions (rather than their general solutions). To this end, a numerical example was conducted where a peripherally fixed circular membrane with Poisson’s ratio *v* = 0.45, Young’s modulus of elasticity *E* = 3.05 MPa, thickness *h* = 0.3 mm and radius *a* = 70 mm was subjected to the weight of the liquid with density *ρ* = 1 × 10^−6^ kg/mm^3^ and height *H* = 300 mm. For convenience, the infinite power series in Equations (26) and (27) have to be truncated to *n* terms, that is

(29) $$(1-\nu )\sum_{i=0}^{n}c_{i}+\sum_{i=1}^{n}ic_{i}=0$$

and

(30) $$\sum_{i=0}^{n}d_{i}=0.$$

The value of the parameter *n* in Equations (29) and (30) should be specified firstly, and then all the recursion formulas for the coefficients *c_i_* and *d_i_* in Appendix A and Appendix B are repeatedly substituted into Equations (29) and (30) until a system of equations containing only the undetermined constants *c*_0_ and *d*_0_ can be finally obtained. The numerical values of *c*_0_ and *d*_0_, which correspond to the specified value of the parameter *n*, can be determined by solving this system of equations with regard to *c*_0_ and *d*_0_.

**Figure 3:**
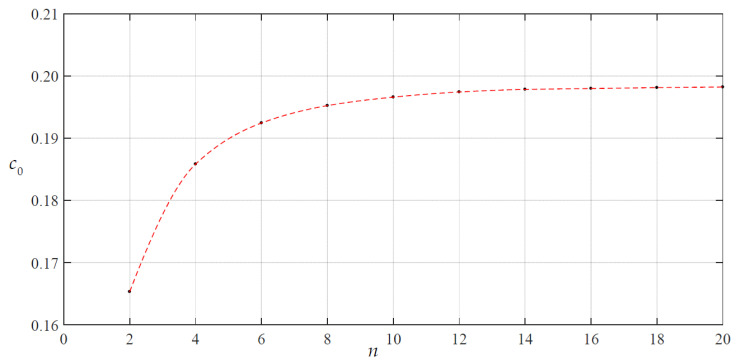
Variation of *c*_0_ with *n* when *H* = 300 mm.

**Figure 4:**
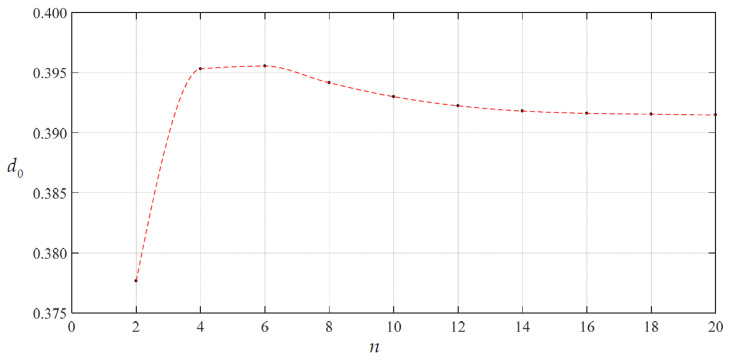
Variation of *d*_0_ with *n* when *H* = 300 mm.

We began the numerical value calculations of *c*_0_ and *d*_0_ from *n* = 2; the calculation results are listed in Table 1 and the variations of *c*_0_ and *d*_0_ with *n* are shown in Figure 3 and Figure 4. From Figure 3 and Figure 4, it may be seen that the data sequences of *c*_0_ and *d*_0_ already converge well when *n* = 18. Therefore, only the recursion formulas for the coefficients *c_i_* and *d_i_* when *i* ≤ 20 are listed in Appendix A and Appendix B, and the undetermined constants *c*_0_ and *d*_0_ can finally take 1.98216876 × 10^−1^ and 3.91482802 × 10^−1^, respectively, i.e., the values at *n* = 20 in Table 1.

**Figure 5:**
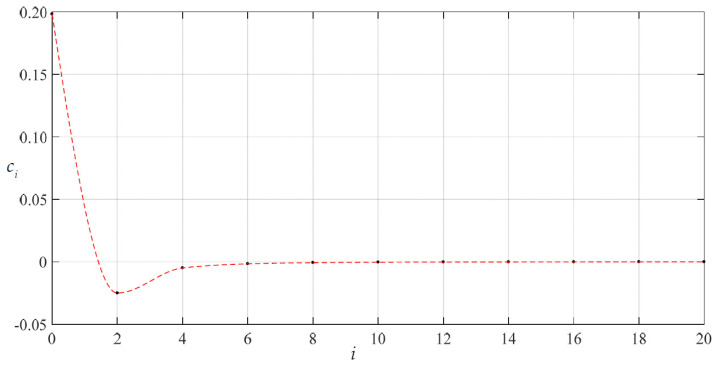
Variation of *c_i_* with *i* when *c*_0_ = 1.98216876 × 10^−1^, *d*_0_ = 3.91482802 × 10^−1^ and *H* = 300 mm.

**Figure 6:**
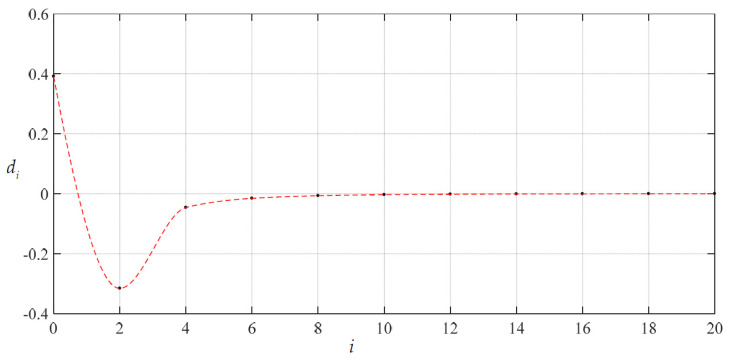
Variation of *d_i_* with *i* when *c*_0_ = 1.98216876 × 10^−1^, *d*_0_ = 3.91482802 × 10^−1^ and *H* = 300 mm.

To examine the convergence of the special solutions of stress and deflection with *c*_0_ = 1.98216876 × 10^−1^ and *d*_0_ = 3.91482802 × 10^−1^ the numerical values of *c_i_* and *d_i_* were calculated, as listed in Table 2. The variations of *c_i_* and *d_i_* with *i* are shown in Figure 5 and Figure 6. It may be seen from Figure 5 and Figure 6 that the special solutions of stress and deflection at *x* = 1 (i.e., at *r* = *a* = 70 mm, the worst case) converge very well.

### 3.2. The Improved Effect of the Integro-Differential Out-of-Plane Equilibrium Equation

**Figure 7:**
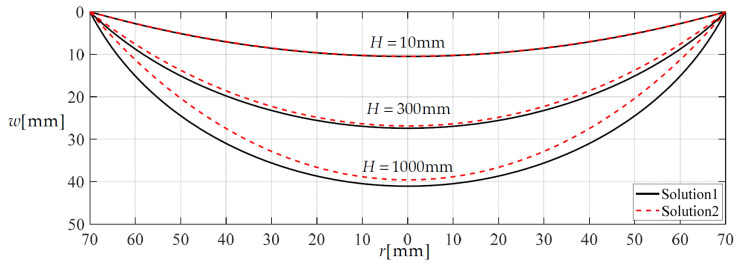
The variations of *w* with *r* when *H* takes 10, 300 and 1000 mm, respectively.

**Figure 8:**
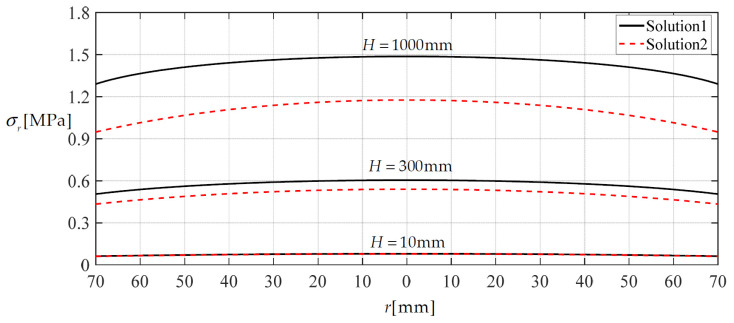
Variations of *σ_r_* with *r* when *H* is 10, 300 and 1000 mm, respectively.

In Section 2, the approximation of replacing sin*θ* = 1/(1 + 1/tan^2^*θ*)^1/2^ with sin*θ* = tan*θ* has been discarded during the derivation of the integro-differential equation for governing the out-of-plane equilibrium. Now, let us see the effect of giving up this approximation on the closed-form solutions. Figure 7 and Figure 8 show the variation of deflection and stress along the diameter when the height *H* of the rainwater stored in the container reaches 10, 300 and 1000 mm, respectively. In Figure 7 and Figure 8, Solution 1 refers to the closed-form solution, which is obtained by using sin*θ* = 1/(1 + 1/tan^2^*θ*)^1/2^ in Section 2, while Solution 2 refers to the closed-form solution which is obtained by using sin*θ* = tan*θ* in [5]. Therefore, the comparison between Solution 1 and Solution 2 can reflect the effect of giving up the approximation of replacing sin*θ* = 1/(1 + 1/tan^2^*θ*)^1/2^ with sin*θ* = tan*θ*. It may be seen from Figure 7 and Figure 8 that the two solutions agree quite closely when the height *H* of the rainwater in the storage container is relatively small, but as the height *H* increases they gradually diverge. This means that the use of sin*θ* = 1/(1 + 1/tan^2^*θ*)^1/2^ in Solution 1 has a noticeable effect.

### 3.3. Two Typical Applications of the Closed-form Solution Given

**Figure 9:**
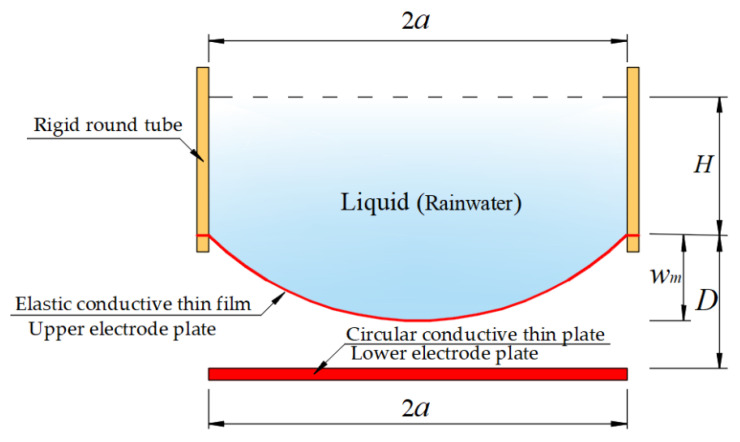
Sketch of rain gauges based on membrane deflection and non-parallel plate capacitor.

The membrane deflection-based rain gauges to be developed will involve two main types: one directly measures the maximum deflection *w*_m_ of the deflected circular membrane, as shown in Figure 1, and the other is to measure the capacitance of the non-parallel plate capacitor as shown in Figure 9. The first type of rain gauge can use any thin film with good elasticity as the elastic bottom of the rainwater storage container, while the second type must use conductive thin films [30,31] with both good elasticity and good electrical conductivity as the upper electrode plate of the non-parallel plate capacitor (see Figure 9). Let us continue with the numerical examples conducted in Section 3.1 to illustrate the application of the closed-form solution given in Section 2 in designing such membrane deflection-based rain gauges. We present here only the numerical calibration of such rain gauges based on the closed-form solution given in Section 2.

**Figure 10:**
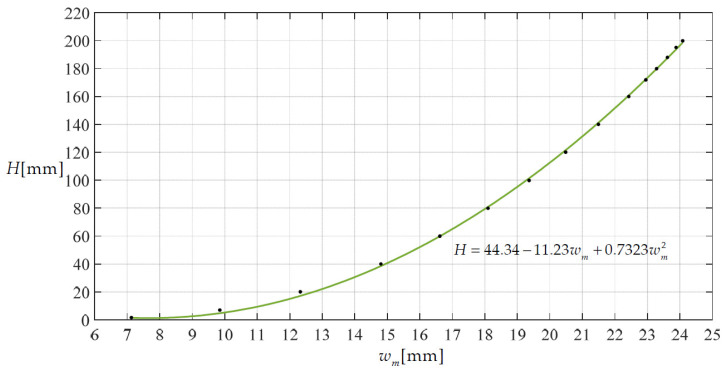
A scatter diagram describing the relationship of height *H* and maximum deflection *w*_m_.

For the first type of rain gauge, the maximum deflection *w*_m_ of the deflected circular membrane can be directly measured, for example, by a non-contact laser displacement sensor. Figure 10 shows a scatter diagram describing the relationship between the height *H* of the rainwater in the storage container and the maximum deflection *w*_m_ of the deflected circular membrane, where the values of the scatter points are calculated using the closed-form solution given in Section 2, and then to fit the curve *H* = 44.34 − 11.23 *w*_m_ + 0.7323 *w*_m_^2^. Therefore, with the measured values of the maximum deflection *w*_m_, the corresponding values of the height *H* of the rainwater in the storage container can be determined using the analytical expression *H* = 44.34 − 11.23 *w*_m_ + 0.7323 *w*_m_^2^.

As for the second type of rain gauge shown in Figure 9, the capacitance of the non-parallel plate capacitor is given by [13,32].

(31) $$C=\varepsilon_{0}\varepsilon_{r}\int_{0}^{2\pi }\int_{0}^{a}\frac{r}{D-w(r,\varphi )}d\varphi dr=2\pi \varepsilon_{0}\varepsilon_{r}\int_{0}^{a}\frac{r}{D-w(r)}dr,$$

where *ε*_0_ is the vacuum dielectric constant (*ε*_0_ = 8.854187817 × 10^−12^ F/m), *ε*_r_ is the relative permittivity of dry air (*ε*_r_ = 1.000585), and *D* is the initial gap between the initial flat circular membrane and the circular conductive thin plate (suppose that *D* takes 35 mm here). From Equations (17) and (25) the dimensional deflection *w*(*r*) can be written as

(32) $$w(r)=\sum_{i=0}^{\infty }\frac{d_{i}}{a^{i-1}}r^{i}.$$

If letting

(33) $$\frac{r}{D-w(r)}=\sum_{i=0}^{\infty }b_{i}r^{i},$$

then it is not difficult that the coefficients *b_i_* is expressed as the polynomials with regards to *d*_i_ and *D*. Therefore, the capacitance of the non-parallel plate capacitor as shown in Figure 9 can be finally written as

(34) $$C=2\pi \varepsilon_{0}\varepsilon_{r}\int_{0}^{a}(\sum_{i=0}^{\infty }b_{i}r^{i})dr=2\pi \varepsilon_{0}\varepsilon_{r}\sum_{i=0}^{\infty }\frac{b_{i}a^{i+1}}{i+1},$$

where *b*_i_ ≡ 0 (*i* = 0, 2, 4, …) and *b*_i_ (*i* = 1, 3, 5, …) are listed in Appendix C.

**Figure 11:**
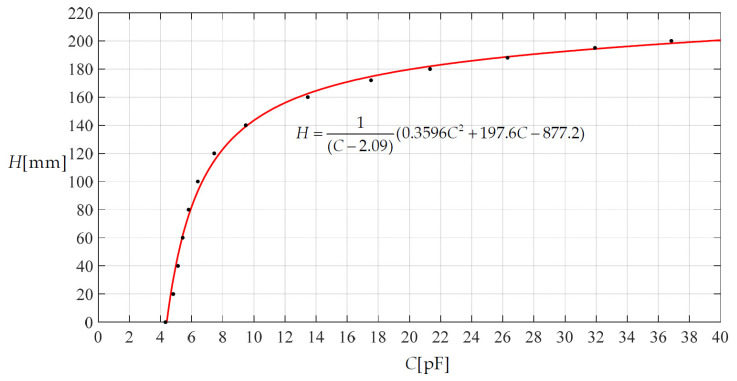
A scatter diagram describing the relationship of height *H* and capacitance *C*.

Figure 11 shows a scatter diagram describing the relationship between the height *H* of the rainwater in the storage container and the capacitance *C* of the non-parallel plate capacitor, where the values of the scatter points are calculated using the closed-form solution given in Section 2 and Equation (34), and then used to fit the curve *H* = (0.3596*C*^2^ + 197.6*C* − 877.2)/(*C* − 2.09). Therefore, with the measured values of the capacitance *C*, the corresponding values of the height *H* of the rainwater in the storage container can be determined using the analytical expression *H* = (0.3596*C*^2^ + 197.6*C* ‒ 877.2)/(*C* − 2.09).

### 3.4. Confirmatory Experiment

**Figure 12:**
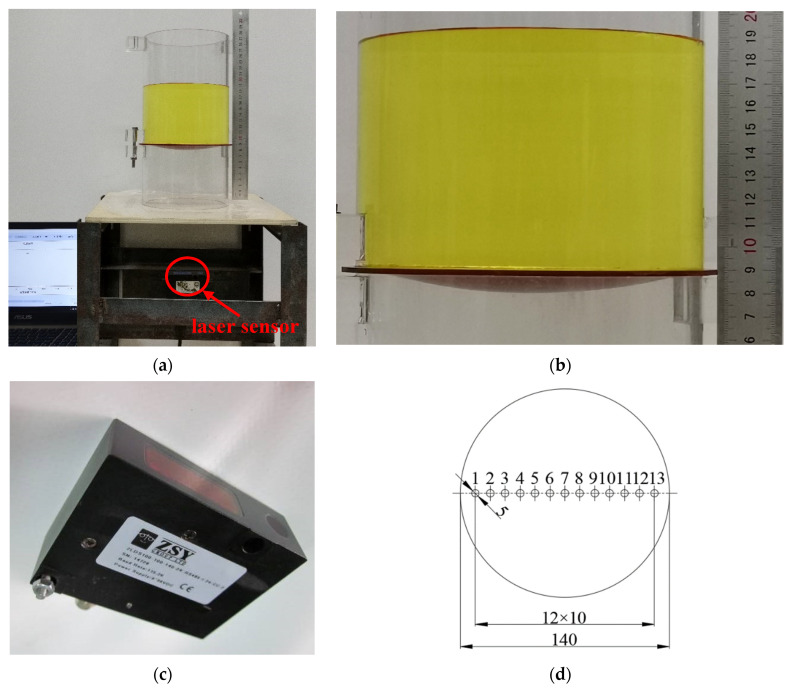
Confirmatory experiment: (**a**) experimental setup; (**b**) a detailed view of the colored water part (**a**); (**c**) laser displacement sensor; (**d**) positions of 13 test points.

**Figure 13:**
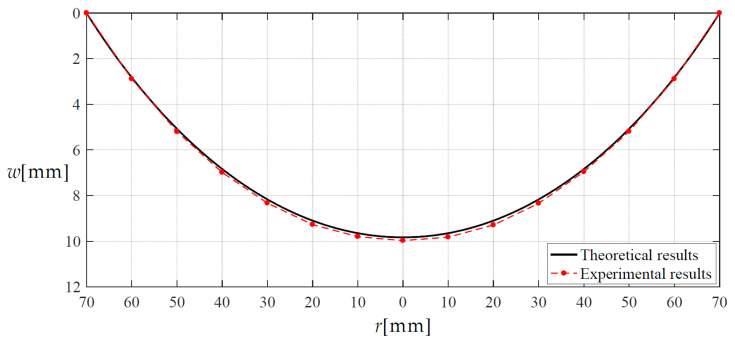
Membrane deflection along the diameter when *H* = 97.5 mm.

In order to verify the validity of the closed-form solution given in Section 2, we conducted a confirmatory experiment. As shown in Figure 12, a peripherally fixed circular silicon rubber thin-film with Poisson’s ratio *v* = 0.45, Young’s modulus of elasticity *E* = 3.05 MPa, thickness *h* = 2 mm and radius *a* = 70 mm was subjected to the weight of the liquid (colored water) with density *ρ* = 1 × 10^−6^ kg/mm^3^ and height *H* = 100 mm. We use a non-contact laser displacement sensor (ZSY Group Ltd., London, UK, see Figure 12c) to measure membrane deflection at 13 test points (see Figure 12d). The results of the experimental test and theoretical calculation of deflection as well as their relative errors are listed in Table 3, and the deflection curves along the diameter are shown in Figure 13.

It may be seen from Table 3 or Figure 13 that the results of experimental test and theoretical calculation agree well, which means that the closed-form solution obtained in Section 2 is basically reliable. Of course, the computational accuracy of the closed-form solution presented here needs to be further improved. Some approximations or assumptions are still used during the derivation of the in-plane equilibrium and geometric equations, which should be the main reason for the relative errors in Table 3.

## 4. Concluding Remarks

In this paper, the statics problem of the fluid–structure interaction of a peripherally fixed circular membrane subjected to liquid weight loading is reformulated, where the approximation of replacing sin*θ* = 1/(1 + 1/tan^2^*θ*)^1/2^ with sin*θ* = tan*θ*, which is adopted in the earlier work [5], is discarded. The previous integro-differential equation governing the out-of-plane equilibrium, established by using sin*θ* = tan*θ* in [5], was modified using sin*θ* = 1/(1 + 1/tan^2^*θ*)^1/2^, resulting in a new and more complicated integro-differential equation governing the out-of-plane equilibrium. The reformulated problem was solved using the power series method, and a new and more refined closed-form solution of the problem was finally given. Some important issues were addressed numerically and experimentally. The following conclusions can be drawn from this study.

The sine function, sin*θ*, can be approximated by the tangent function, tan*θ*, only when the rotation angle *θ* of membrane is relatively small; when the rotation angle *θ* of membrane is relatively large, such an approximation will give rise to a significant error. For instance, the error caused by using tan*θ* to approximate sin*θ* was about 1.54% when *θ* = 10°, 6.42% when *θ* = 20°, 15.47% when *θ* = 30°, and 30.54% when *θ* = 40°. In fact, the rotation-angle *θ* of the membrane may exceed 40° for membrane deflection-based rain gauges to be developed. Therefore, it is necessary and worthwhile for such technical applications to discard the approximation of replacing sin*θ* = 1/(1 + 1/tan^2^*θ*)^1/2^ with sin*θ* = tan*θ* during the derivation of the closed-form solution of the problem under consideration.

The power series method is a very effective mathematical tool for solving nonlinear equations. Although the new integro-differential equation governing the out-of-plane equilibrium is much more complicated than the previous one, the power series solutions obtained for stress and deflection still have good convergence and fast convergence speed.

In addition, the closed-form solution obtained in Section 2 is in good agreement with the confirmatory experiment conducted, suggesting that this closed-form solution is basically reliable and can be used to design the membrane deflection-based rain gauges to be developed.

## Figures and Tables

**Table 1:** Numerical values of *c*_0_ and *d*_0_ at different *n* when *H* = 300 mm.

*n*	*c*_0_	*d*_0_
2	1.65316391 × 10^−1^	3.77658452 × 10^−1^
4	1.85832249 × 10^−1^	3.95315235 × 10^−1^
6	1.92438471 × 10^−1^	3.95548206 × 10^−1^
8	1.95231453 × 10^−1^	3.94159221 × 10^−1^
10	1.96595308 × 10^−1^	3.93001167 × 10^−1^
12	1.97422893 × 10^−1^	3.92241509 × 10^−1^
14	1.97834610 × 10^−1^	3.91803156 × 10^−1^
16	1.97977443 × 10^−1^	3.91614348 × 10^−1^
18	1.98125067 × 10^−1^	3.91540206 × 10^−1^
20	1.98216876 × 10^−1^	3.91482802 × 10^−1^

**Table 2:** Numerical values of *c_i_* and *d_i_* when *c*_0_ = 1.98216876 × 10^−1^, *d*_0_ = 3.91482802 × 10^−1^ and *H* = 300 mm.

*i*	*c_i_*	*d_i_*
0	1.98216876 × 10^−1^	3.91482802 × 10^−1^
2	−2.49493987 × 10^−2^	−3.15907573 × 10^−1^
4	−4.85171579 × 10^−3^	−4.60740692 × 10^−2^
6	−1.58206170 × 10^−3^	−1.55521234 × 10^−2^
8	−6.42288769 × 10^−4^	−6.76343212 × 10^−3^
10	−2.94655565 × 10^−4^	−3.32901660 × 10^−3^
12	−1.46220841 × 10^−4^	−1.76487967 × 10^−3^
14	−7.66573427 × 10^−5^	−9.83324535 × 10^−4^
16	−4.18541507 × 10^−5^	−5.67748867 × 10^−4^
18	−2.35781462 × 10^−5^	−3.36715864 × 10^−4^
20	−1.36166630 × 10^−5^	−2.03918152 × 10^−4^

**Table 3:** Experimental and theoretical values of deflection and their relative errors when *H* = 97.5 mm.

Test Points	Radius	Experimental Results	Calculated Results	Relative Errors
1	60	2.89	2.8400	1.73%
2	50	5.19	5.1016	1.70%
3	40	6.99	6.8729	1.67%
4	30	8.33	8.2082	1.46%
5	20	9.27	9.1414	1.39%
6	10	9.80	9.6937	1.08%
7	0	9.97	9.8766	0.94%
8	10	9.82	9.6937	1.29%
9	20	9.29	9.1414	1.60%
10	30	8.34	8.2082	1.58%
11	40	6.96	6.8729	1.25%
12	50	5.19	5.1016	1.70%
13	60	2.89	2.8400	1.73%

Relative errors = |Experimental results–Theoretical results|/Experimental results.

## Data Availability

Not applicable.

## Nomenclature

*a*	Radius of the circular membrane
*h*	Thickness of the circular membrane
*E*	Young’s modulus of elasticity
*ν*	Poisson’s ratio
*H*	Height of the liquid in the storage container
*ρ*	Density of the liquid in the storage container
*g*	Acceleration of gravity
*r*	Radial coordinate of the cylindrical coordinate system (*r*, *φ*, *w*)
*φ*	Circumferential angle coordinate of (*r*, *φ*, *w*)
*w*	Axial coordinate of (*r*, *φ*, *w*) as well as transverse displacement
*u*	Radial displacement of the deflected circular membrane
*w*_m_	Maximum deflection of the deflected circular membrane
*q*(*r*)	Transverse loads that varies continuously with the radial coordinate *r*
*F*(*r*)	External force produced by *q*(*r*) within radius *r*
*σ*_*r*_	Radial stress
*σ*_*t*_	Circumferential stress
*e*_*r*_	Radial strain
*e*_*t*_	Circumferential strain
*θ*	Rotation angle of the deflected circular membrane
*π*	Pi (ratio of circumference to diameter)
*W*	Dimensionless transverse displacement (*w*/*a*)
*S*_*r*_	Dimensionless radial stress (*σ_r_*/*E*)
*S*_*t*_	Dimensionless circumferential stress (*σ_t_*/*E*)
*H*_0_	Dimensionless height *H* (*H*/*a*)
*G*	Dimensionless quantity (*ρga*^2^/*Eh*)
*x*	Dimensionless radial coordinate (*r*/*a*)
*C*	Capacitance of a non-parallel plate capacitor
*D*	Initial gap of the non-parallel plate capacitor
*b*_*i*_	Coefficients of the power series for capacitance *C*
*c*_*i*_	Coefficients of the power series for *S_r_*
*d*_*i*_	Coefficients of the power series for *W*

## Appendix A

$$c_{2}=-\frac{G^{2}{\left(H_{0}+d_{0}\right)}^{2}}{64c_{0}^{2}},$$

$$c_{4}=-\frac{1}{192\;c_{0}^{2}}(4\;G^{2}H_{0}^{2}d_{2}^{2}+8\;G^{2}H_{0}d_{0}d_{2}^{2}+4\;G^{2}d_{0}^{2}d_{2}^{2}+G^{2}H_{0}d_{2}+G^{2}d_{0}d_{2}-32\;c_{0}c_{2}d_{2}^{2}),$$

$$\begin{array}{l} c_{6}=-\frac{1}{4608\;c_{0}^{2}}(192\;G^{2}H_{0}^{2}d_{2}d_{4}+384\;G^{2}H_{0}d_{0}d_{2}d_{4}+48\;G^{2}H_{0}d_{2}^{3}+192\;G^{2}d_{0}^{2}d_{2}d_{4}+48\;G^{2}d_{0}d_{2}^{3}\\ \;+8\;G^{2}H_{0}d_{4}+8\;G^{2}d_{4}d_{0}+3\;G^{2}d_{2}^{2}-1536\;c_{0}c_{2}d_{2}d_{4}-384\;c_{0}c_{4}d_{2}^{2}-192\;c_{2}^{2}d_{2}^{2}) \end{array}$$

$$\begin{array}{l} c_{8}=-\frac{1}{3840\;c_{0}^{2}}(144\;G^{2}H_{0}^{2}d_{2}d_{6}+96\;G^{2}H_{0}^{2}d_{4}^{2}+288\;G^{2}H_{0}d_{0}d_{2}d_{6}+192\;G^{2}H_{0}d_{0}d_{4}^{2}+96\;G^{2}d_{0}^{2}d_{4}^{2}\\ \;+112\;G^{2}H_{0}d_{2}^{2}d_{4}+144\;G^{2}d_{0}^{2}d_{2}d_{6}+112\;G^{2}d_{0}d_{0}^{2}d_{4}+6\;G^{2}d_{2}^{4}+3\;G^{2}H_{0}d_{6}-768\;c_{0}c_{4}d_{2}d_{4}\\ \;+3\;G^{2}d_{6}d_{0}+2\;G^{2}d_{4}d_{2}-1152\;c_{0}c_{2}d_{2}d_{6}-768\;c_{0}c_{2}d_{4}^{2}-192\;c_{0}c_{6}d_{2}^{2}-384\;c_{2}^{2}d_{2}d_{4}-192\;c_{2}c_{4}d_{2}^{2}) \end{array}$$

$$\begin{array}{l} c_{10}=-\frac{1}{172800c_{0}^{2}}(5760G^{2}H_{0}^{2}d_{2}d_{8}+8640G^{2}H_{0}^{2}d_{4}d_{6}+11520G^{2}H_{0}d_{0}d_{2}d_{8}+17280G^{2}H_{0}d_{0}d_{4}d_{6}\\ \;+4680G^{2}H_{0}d_{2}^{2}d_{6}+4800G^{2}H_{0}d_{2}d_{4}^{2}+5760G^{2}d_{0}^{2}d_{2}d_{8}+8640G^{2}d_{0}^{2}d_{4}d_{6}+4680G^{2}d_{0}d_{2}^{2}d_{6}\\ \;+4800G^{2}d_{0}d_{2}d_{4}^{2}+960G^{2}d_{2}^{3}d_{4}+72G^{2}H_{0}d_{8}+72G^{2}d_{0}d_{8}+45G^{2}d_{2}d_{6}+20G^{2}d_{4}^{2}\\ \;-46080c_{0}c_{2}d_{2}d_{8}-69120c_{0}c_{2}d_{4}d_{6}-34560c_{0}c_{4}d_{2}d_{6}-23040c_{0}c_{4}d_{4}^{2}-23040c_{0}c_{6}d_{2}d_{4}\\ \;-5760c_{0}c_{8}d_{2}^{2}-17280c_{2}^{2}d_{2}d_{6}-11520c_{2}^{2}d_{4}^{2}-23040c_{2}c_{4}d_{2}d_{4}-5760c_{{}_{2}}c_{6}d_{2}^{2}-2880c_{4}^{2}d_{2}^{2}) \end{array}$$

$$\begin{array}{l} c_{12}=-\frac{1}{120960c_{0}^{2}}(3600G^{2}H_{0}^{2}d_{2}d_{10}+5760G^{2}H_{0}^{2}d_{4}d_{8}+3240G^{2}H_{0}^{2}d_{6}^{2}+7200G^{2}H_{0}d_{0}d_{2}d_{10}\\ \;+11520G^{2}H_{0}d_{0}d_{4}d_{8}+6480G^{2}H_{0}d_{0}d_{6}^{2}+3024G^{2}H_{0}d_{2}^{2}d_{8}+6480G^{2}H_{0}d_{2}d_{4}d_{6}+960G^{2}H_{0}d_{4}^{3}\\ \;+3600G^{2}d_{0}^{2}d_{2}d_{10}+5760G^{2}d_{0}^{2}d_{4}d_{8}+3240G^{2}d_{0}^{2}d_{6}^{2}+3024G^{2}d_{0}d_{2}^{2}d_{8}+6480G^{2}d_{0}d_{2}d_{4}d_{6}\\ \;+960G^{2}d_{0}d_{4}^{3}+630G^{2}d_{2}^{3}d_{6}-46080c_{0}c_{2}d_{4}d_{8}+30G^{2}H_{0}d_{10}+30G^{2}d_{0}d_{10}+18G^{2}d_{2}d_{8}\\ \;+15G^{2}d_{4}d_{6}-28800c_{0}c_{2}d_{2}d_{10}+880G^{2}d_{2}^{2}d_{4}^{2}-25920c_{0}c_{2}d_{6}^{2}-23040c_{0}c_{4}d_{2}d_{8}-34560c_{0}c_{4}d_{4}d_{6}\\ \;-17280c_{0}c_{6}d_{2}d_{6}-11520c_{0}c_{6}d_{4}^{2}-11520c_{0}c_{8}d_{2}d_{4}-2880c_{0}c_{10}d_{2}^{2}-11520c_{2}^{2}d_{2}d_{8}-17280c_{2}^{2}d_{4}d_{6}\\ \;-17280c_{2}c_{4}d_{2}d_{6}-11520c_{2}c_{4}d_{4}^{2}-11520c_{0}c_{6}d_{2}d_{4}-2880c_{2}c_{8}d_{2}^{2}-5760c_{4}^{2}d_{2}d_{4}-2880c_{4}c_{6}d_{2}^{2}) \end{array}$$

$$\begin{array}{l} c_{14}=-\frac{1}{9031680c_{0}^{2}}(241920G^{2}H_{0}^{2}d_{2}d_{12}+403200G^{2}H_{0}^{2}d_{4}d_{10}+483840G^{2}H_{0}^{2}d_{6}d_{8}-322560c_{4}^{2}d_{4}^{2}\\ \;+483840G^{2}H_{0}d_{0}d_{2}d_{12}+806400G^{2}H_{0}d_{0}d_{4}d_{10}+967680G^{2}H_{0}d_{0}d_{6}d_{8}+208320G^{2}H_{0}d_{2}^{2}d_{10}\\ \;+462336G^{2}H_{0}d_{2}d_{4}d_{8}+241920G^{2}H_{0}d_{2}d_{6}^{2}+201600G^{2}H_{0}d_{4}^{2}d_{6}+241920G^{2}d_{0}^{2}d_{2}d_{12}\\ \;+403200G^{2}d_{0}^{2}d_{4}d_{10}+483840G^{2}d_{0}^{2}d_{6}d_{8}+208320G^{2}d_{0}d_{2}^{2}d_{10}+462336G^{2}d_{0}d_{2}d_{4}d_{8}\\ \;+241920G^{2}d_{0}d_{2}d_{6}^{2}+201600G^{2}d_{0}d_{4}^{2}d_{6}+44352G^{2}d_{2}^{3}d_{8}+124320G^{2}d_{2}^{2}d_{4}d_{6}+35840G^{2}d_{2}d_{4}^{3}\\ \;+1440G^{2}H_{0}d_{12}+1440G^{2}d_{0}d_{12}+840G^{2}d_{2}d_{10}+672G^{2}d_{4}d_{8}+315G^{2}d_{6}^{2}-1935360c_{0}c_{2}d_{2}d_{12}\\ \;-3225600c_{0}c_{2}d_{4}d_{10}-3870720c_{0}c_{2}d_{6}d_{8}-1612800c_{0}c_{4}d_{2}d_{10}-2580480c_{0}c_{4}d_{4}d_{8}\\ \;-1451520c_{0}c_{4}d_{6}^{2}-1290240c_{0}c_{6}d_{2}d_{8}-1935360c_{0}c_{6}d_{4}d_{6}-967680c_{0}c_{8}d_{2}d_{6}-645120c_{0}c_{8}d_{4}^{2}\\ \;-645120c_{0}c_{10}d_{2}d_{4}-161280c_{0}c_{12}d_{2}^{2}-806400c_{2}^{2}d_{2}d_{10}-1290240c_{2}^{2}d_{4}d_{8}-725760c_{2}^{2}d_{6}^{2}\\ \;-1290240c_{2}c_{4}d_{2}d_{8}-1935360c_{2}c_{4}d_{4}d_{6}-967680c_{2}c_{6}d_{2}d_{6}-645120c_{2}c_{6}d_{4}^{2}-645120c_{2}c_{8}d_{2}d_{4}\\ \;-161280c_{2}c_{10}d_{2}^{2}-483840c_{4}^{2}d_{2}d_{6}-645120c_{4}c_{6}d_{2}d_{4}-161280c_{4}c_{8}d_{2}^{2}-80640c_{6}^{2}d_{2}^{2})\; \end{array}$$

$$\begin{array}{l} c_{16}=-\frac{1}{2903040c_{0}^{2}}(70560\;G^{2}H_{0}^{2}d_{2}d_{14}+120960\;G^{2}H_{0}^{2}d_{4}d_{12}+151200\;G^{2}H_{0}^{2}d_{6}d_{10}+80640\;G^{2}H_{0}^{2}d_{8}^{2}\\ \;+141120\;G^{2}H_{0}d_{0}d_{2}d_{14}+241920\;G^{2}H_{0}d_{0}d_{4}d_{12}+302400\;G^{2}H_{0}d_{0}d_{6}d_{10}+161280\;G^{2}H_{0}d_{0}d_{8}^{2}\\ \;+61920\;G^{2}H_{0}d_{2}^{2}d_{12}+141120\;G^{2}H_{0}d_{2}d_{4}d_{10}+153216\;G^{2}H_{0}d_{2}d_{6}d_{8}+61824\;G^{2}H_{0}d_{4}^{2}d_{8}\\ \;+60480\;G^{2}H_{0}d_{4}d_{6}^{2}+70560\;G^{2}d_{0}^{2}d_{2}d_{14}+120960\;G^{2}d_{0}^{2}d_{4}d_{12}+151200\;G^{2}d_{0}^{2}d_{6}d_{10}\\ \;+80640\;G^{2}d_{0}^{2}d_{8}^{2}+61920\;G^{2}d_{0}d_{2}^{2}d_{12}+141120\;G^{2}d_{0}d_{2}d_{4}d_{10}+153216\;G^{2}d_{0}d_{2}d_{6}d_{8}\\ \;+61824\;G^{2}d_{0}d_{4}^{2}d_{8}+60480\;G^{2}d_{0}d_{4}d_{6}^{2}+13440\;G^{2}d_{2}^{3}d_{10}+38304\;G^{2}d_{2}^{2}d_{4}d_{8}+140\;G^{2}d_{4}d_{10}\\ \;+19215\;G^{2}d_{2}^{2}d_{6}{}^{2}+31920\;G^{2}d_{2}d_{4}^{2}d_{6}+2240\;G^{2}d_{4}^{4}+315\;G^{2}H_{0}d_{14}+315\;G^{2}d_{0}d_{14}+180\;G^{2}d_{2}d_{12}\\ \;+126\;G^{2}d_{6}d_{8}-564480\;c_{0}c_{2}d_{2}d_{14}-967680\;c_{0}c_{2}d_{4}d_{12}-1209600\;c_{0}c_{2}d_{6}d_{10}-645120\;c_{0}c_{2}d_{8}^{2}\\ \;-806400\;c_{0}c_{4}d_{4}d_{10}-967680\;c_{0}c_{4}d_{6}d_{8}-403200\;c_{0}c_{6}d_{2}d_{10}-645120\;c_{0}c_{6}d_{4}d_{8}-483840\;c_{0}c_{4}d_{2}d_{12}\\ \;-362880\;c_{0}c_{6}d_{6}^{2}-322560\;c_{0}c_{8}d_{2}d_{8}-483840\;c_{0}c_{8}d_{4}d_{6}-241920\;c_{0}c_{10}d_{2}d_{6}-161280\;c_{0}c_{10}d_{4}^{2}\\ \;-161280\;c_{0}c_{12}d_{2}d_{4}-40320\;c_{0}c_{14}d_{2}^{2}-241920\;c_{2}^{2}d_{2}d_{12}-403200\;c_{2}^{2}d_{4}d_{10}-483840\;c_{2}^{2}d_{6}d_{8}\\ \;-403200\;c_{2}c_{4}d_{2}d_{10}-645120\;c_{2}c_{4}d_{4}d_{8}-362880\;c_{2}c_{4}d_{6}^{2}-322560\;c_{2}c_{6}d_{2}d_{8}-483840\;c_{2}c_{6}d_{4}d_{6}\\ \;-241920\;c_{2}c_{8}d_{2}d_{6}-161280\;c_{2}c_{8}d_{4}^{2}-161280\;c_{2}c_{10}d_{2}d_{4}-40320\;c_{2}c_{12}d_{2}^{2}-161280\;c_{4}{}^{2}d_{2}d_{8}\\ \;-241920\;c_{4}^{2}d_{4}d_{6}-241920\;c_{4}c_{6}d_{2}d_{6}-161280\;c_{4}c_{6}d_{4}^{2}-161280\;c_{4}c_{8}d_{2}d_{4}-40320\;c_{4}c_{10}d_{2}^{2}\\ \;-80640\;c_{6}^{2}d_{2}d_{4}-40320\;c_{6}c_{8}d_{2}^{2}) \end{array}$$

$$\begin{array}{l} c_{18}=-\frac{1}{36288000c_{0}^{2}}(806400\;G^{2}H_{0}^{2}d_{2}d_{16}+1411200\;G^{2}H_{0}^{2}d_{4}d_{14}+1814400\;G^{2}H_{0}^{2}d_{6}d_{12}\\ \;+2016000\;G^{2}H_{0}^{2}d_{8}d_{10}+1612800\;G^{2}H_{0}d_{0}d_{2}d_{16}+2822400\;G^{2}H_{0}d_{0}d_{4}d_{14}+718200\;d_{2}^{2}G^{2}d_{14}H_{0}\\ \;+4032000\;G^{2}H_{0}d_{0}d_{8}d_{10}+3628800\;G^{2}H_{0}d_{0}d_{6}d_{12}+1670400\;G^{2}H_{0}d_{2}d_{4}d_{12}+967680\;G^{2}H_{0}d_{2}d_{8}^{2}\\ \;+1864800\;G^{2}H_{0}d_{2}d_{6}d_{10}+739200\;G^{2}H_{0}d_{4}^{2}d_{10}+1451520\;G^{2}H_{0}d_{4}d_{6}d_{8}+226800\;G^{2}H_{0}d_{6}^{3}\\ \;+806400\;G^{2}d_{0}^{2}d_{2}d_{16}+1411200\;G^{2}d_{0}^{2}d_{4}d_{14}+1814400\;G^{2}d_{0}^{2}d_{6}d_{12}+2016000\;G^{2}d_{0}^{2}d_{8}d_{10}\\ \;+718200\;d_{2}^{2}G^{2}d_{14}d_{0}+1670400\;G^{2}d_{0}d_{2}d_{4}d_{12}+1864800\;G^{2}d_{0}d_{2}d_{6}d_{10}+967680\;G^{2}d_{0}d_{2}d_{8}^{2}\\ \;+739200\;G^{2}d_{0}d_{4}^{2}d_{10}+1451520\;G^{2}d_{0}d_{4}d_{6}d_{8}+226800\;G^{2}d_{0}d_{6}^{3}+158400\;G^{2}d_{2}^{3}d_{12}+1050\;G^{2}d_{6}d_{10}\\ \;+459200\;G^{2}d_{2}^{2}d_{4}d_{10}+468720\;G^{2}d_{2}^{2}d_{6}d_{8}+380800\;G^{2}d_{2}d_{4}^{2}d_{8}+365400\;G^{2}d_{2}d_{4}d_{6}^{2}+504\;G^{2}d_{8}^{2}\\ \;+100800\;G^{2}d_{4}^{3}d_{6}+2800\;G^{2}d_{16}H_{0}+2800\;G^{2}d_{16}d_{0}+1575\;G^{2}d_{2}d_{14}+1200\;G^{2}d_{4}d_{12}\\ \;-6451200\;c_{0}c_{2}d_{2}d_{16}-11289600\;c_{0}c_{2}d_{4}d_{14}-14515200\;c_{0}c_{2}d_{6}d_{12}-16128000\;c_{0}c_{2}d_{8}d_{10}\\ \;-5644800\;c_{0}c_{4}d_{2}d_{14}-9676800\;c_{0}c_{4}d_{4}d_{12}-12096000\;c_{0}c_{4}d_{6}d_{10}-6451200\;c_{0}c_{4}d_{8}^{2}\\ \;-4838400\;c_{0}c_{6}d_{2}d_{12}-8064000\;c_{0}c_{6}d_{4}d_{10}-9676800\;c_{0}c_{6}d_{6}d_{8}-4032000\;c_{0}c_{8}d_{2}d_{10}\\ \;-6451200\;c_{0}c_{8}d_{4}d_{8}-3628800\;c_{0}c_{8}d_{6}^{2}-3225600\;c_{0}c_{10}d_{2}d_{8}-4838400\;c_{0}c_{10}d_{4}d_{6}\\ \;-2419200\;c_{0}c_{12}d_{2}d_{6}-1612800\;c_{0}c_{12}d_{4}^{2}-1612800\;d_{2}d_{4}c_{0}c_{14}-403200\;d_{2}^{2}c_{0}c_{16}\\ \;-2822400\;c_{2}^{2}d_{2}d_{14}-4838400\;c_{2}^{2}d_{4}d_{12}-6048000\;c_{2}^{2}d_{6}d_{10}-3225600\;c_{2}^{2}d_{8}^{2}-403200\;d_{2}^{2}c_{6}c_{10}\\ \;-4838400\;c_{2}c_{4}d_{2}d_{12}-8064000\;c_{2}c_{4}d_{4}d_{10}-9676800\;c_{2}c_{4}d_{6}d_{8}-4032000\;c_{2}c_{6}d_{2}d_{10}\\ \;-6451200\;c_{2}c_{6}d_{4}d_{8}-3628800\;c_{2}c_{6}d_{6}^{2}-3225600\;c_{2}c_{8}d_{2}d_{8}-4838400\;c_{2}c_{8}d_{4}d_{6}\\ \;-2419200\;c_{2}c_{10}d_{2}d_{6}-1612800\;c_{2}c_{10}d_{4}^{2}-1612800\;d_{2}d_{4}c_{2}c_{12}-403200\;d_{2}^{2}c_{2}c_{14}\\ \;-2016000\;c_{4}^{2}d_{2}d_{10}-3225600\;c_{4}^{2}d_{4}d_{8}-1814400\;c_{4}^{2}d_{6}^{2}-3225600\;c_{4}c_{6}d_{2}d_{8}\\ \;-4838400\;c_{4}c_{6}d_{4}d_{6}-2419200\;c_{4}c_{8}d_{2}d_{6}-1612800\;c_{4}c_{8}d_{4}^{2}-1612800\;d_{2}d_{4}c_{4}c_{10}\\ \;-403200\;d_{2}^{2}c_{4}c_{12}-1209600\;c_{6}^{2}d_{2}d_{6}-806400\;c_{6}^{2}d_{4}^{2}-1612800\;d_{2}d_{4}c_{6}c_{8}-201600\;d_{2}^{2}c_{8}^{2}) \end{array}$$

$\begin{array}{l} c_{20}=-\frac{1}{22176000c_{0}^{2}}(453600\;G^{2}H_{0}^{2}d_{2}d_{18}+806400\;G^{2}H_{0}^{2}d_{4}d_{16}+1058400\;G^{2}H_{0}^{2}d_{6}d_{14}\\ \;+1209600\;G^{2}H_{0}^{2}d_{8}d_{12}+630000\;G^{2}H_{0}^{2}d_{10}^{2}+907200\;G^{2}H_{0}d_{0}d_{2}d_{18}+1612800\;G^{2}H_{0}d_{0}d_{4}d_{16}\\ \;+2116800\;G^{2}H_{0}d_{0}d_{6}d_{14}+2419200\;G^{2}H_{0}d_{0}d_{8}d_{12}+408800\;G^{2}H_{0}d_{2}^{2}d_{16}+966000\;G^{2}H_{0}d_{2}d_{4}d_{14}\\ \;+1260000\;G^{2}H_{0}d_{0}d_{10}^{2}+1101600\;G^{2}H_{0}d_{2}d_{6}d_{12}+1176000\;G^{2}H_{0}d_{2}d_{8}d_{10}+432000\;G^{2}H_{0}d_{4}^{2}d_{12}\\ \;+856800\;G^{2}H_{0}d_{4}d_{6}d_{10}+430080\;G^{2}H_{0}d_{4}d_{8}^{2}+393120\;G^{2}H_{0}d_{6}^{2}d_{8}+453600\;G^{2}d_{0}^{2}d_{2}d_{18}\\ \;+806400\;G^{2}d_{0}^{2}d_{4}d_{16}+1058400\;G^{2}d_{0}^{2}d_{6}d_{14}+1209600\;G^{2}d_{0}^{2}d_{8}d_{12}+630000\;G^{2}d_{0}^{2}d_{10}^{2}\\ \;+408800\;G^{2}d_{0}d_{2}^{2}d_{16}+966000\;G^{2}d_{0}d_{2}d_{4}d_{14}+1101600\;G^{2}d_{0}d_{2}d_{6}d_{12}+1176000\;G^{2}d_{0}d_{2}d_{8}d_{10}\\ \;+432000\;G^{2}d_{0}d_{4}^{2}d_{12}+856800\;G^{2}d_{0}d_{4}d_{6}d_{10}+430080\;G^{2}d_{0}d_{4}d_{8}^{2}+393120\;G^{2}d_{0}d_{6}^{2}d_{8}\\ \;+91350\;G^{2}d_{2}^{3}d_{14}+268800\;G^{2}d_{2}^{2}d_{4}d_{12}+279300\;G^{2}d_{2}^{2}d_{6}d_{10}+142128\;G^{2}d_{2}^{2}d_{8}^{2}\\ \;+224000\;G^{2}d_{2}d_{4}^{2}d_{10}+426720\;G^{2}d_{2}d_{4}d_{6}d_{8}+66150\;G^{2}d_{2}d_{6}^{3}+58240\;G^{2}d_{4}^{3}d_{8}+81900\;G^{2}d_{4}^{2}d_{6}^{2}\\ \;+1260\;G^{2}H_{0}d_{18}+1260\;G^{2}d_{18}d_{0}+700\;G^{2}d_{16}d_{2}+525\;G^{2}d_{14}d_{4}+450\;G^{2}d_{12}d_{6}+420\;G^{2}d_{10}d_{8}\\ \;-3628800\;c_{0}c_{2}d_{2}d_{18}-6451200\;c_{0}c_{2}d_{4}d_{16}-8467200\;c_{0}c_{2}d_{6}d_{14}-9676800\;c_{0}c_{2}d_{8}d_{12}\\ \;-5040000\;c_{0}c_{2}d_{10}^{2}-3225600\;c_{0}c_{4}d_{2}d_{16}-5644800\;c_{0}c_{4}d_{4}d_{14}-7257600\;c_{0}c_{4}d_{6}d_{12}\\ \;-8064000\;c_{0}c_{4}d_{8}d_{10}-2822400\;c_{0}c_{6}d_{2}d_{14}-4838400\;c_{0}c_{6}d_{4}d_{12}-6048000\;c_{0}c_{6}d_{6}d_{10}\\ \;-3225600\;c_{0}c_{6}d_{8}^{2}-2419200\;c_{0}c_{8}d_{2}d_{12}-4032000\;c_{0}c_{8}d_{4}d_{10}-4838400\;c_{0}c_{8}d_{6}d_{8}\\ \;-2016000\;c_{0}c_{10}d_{2}d_{10}-3225600\;c_{0}c_{10}d_{4}d_{8}-1814400\;c_{0}c_{10}d_{6}^{2}-1612800\;c_{0}c_{12}d_{2}d_{8}\\ \;-2419200\;c_{0}c_{12}d_{4}d_{6}-1209600\;c_{0}c_{14}d_{2}d_{6}-806400\;c_{0}c_{14}d_{4}^{2}-806400\;c_{0}c_{16}d_{2}d_{4}\\ \;-201600\;c_{0}c_{18}d_{2}^{2}-1612800\;c_{2}^{2}d_{2}d_{16}-2822400\;c_{2}^{2}d_{4}d_{14}-3628800\;c_{2}^{2}d_{6}d_{12}\\ \;-4032000\;c_{2}^{2}d_{8}d_{10}-2822400\;c_{2}c_{4}d_{2}d_{14}-4838400\;c_{2}c_{4}d_{4}d_{12}-6048000\;c_{2}c_{4}d_{6}d_{10}\\ \;-3225600\;c_{2}c_{4}d_{8}^{2}-2419200\;c_{2}c_{6}d_{2}d_{12}-4032000\;c_{2}c_{6}d_{4}d_{10}-4838400\;c_{2}c_{6}d_{6}d_{8}\\ \;-2016000\;c_{2}c_{8}d_{2}d_{10}-3225600\;c_{2}c_{8}d_{4}d_{8}-1814400\;c_{2}c_{8}d_{6}^{2}-1612800\;c_{2}c_{10}d_{2}d_{8}\\ \;-2419200\;c_{2}c_{10}d_{4}d_{6}-1209600\;c_{2}c_{12}d_{2}d_{6}-806400\;c_{2}c_{12}d_{4}^{2}-806400\;c_{2}c_{14}d_{2}d_{4}\\ \;-201600\;c_{2}c_{16}d_{2}^{2}-1209600\;c_{4}^{2}d_{2}d_{12}-2016000\;c_{4}^{2}d_{4}d_{10}-2419200\;c_{4}^{2}d_{6}d_{8}\\ \;-2016000\;c_{4}c_{6}d_{2}d_{10}-3225600\;c_{4}c_{6}d_{4}d_{8}-1814400\;c_{4}c_{6}d_{6}^{2}-1612800\;c_{4}c_{8}d_{2}d_{8}\\ \;-2419200\;c_{4}c_{8}d_{4}d_{6}-1209600\;c_{4}c_{10}d_{2}d_{6}-806400\;c_{4}c_{10}d_{4}^{2}-806400\;c_{4}c_{12}d_{2}d_{4}\\ \;-201600\;c_{4}c_{14}d_{2}^{2}-806400\;c_{6}^{2}d_{2}d_{8}-1209600\;c_{6}^{2}d_{4}d_{6}-1209600\;c_{6}c_{8}d_{2}d_{6}-806400\;c_{6}c_{8}d_{4}^{2}\\ \;-806400\;c_{6}c_{10}d_{2}d_{4}-201600\;c_{6}c_{12}d_{2}^{2}-403200\;c_{8}^{2}d_{2}d_{4}-201600\;c_{8}c_{10}d_{2}^{2}) \end{array}$

## Appendix B

$$d_{2}=-\frac{G(H_{0}+d_{0})}{4c_{0}},$$

$$d_{4}=\frac{1}{64\;c_{0}^{2}}(4\;G^{2}H_{0}^{2}d_{2}+8\;G^{2}H_{0}d_{0}d_{2}+4\;G^{2}d_{0}^{2}d_{2}+G^{2}H_{0}+G^{2}d_{0}-32\;c_{0}c_{2}d_{2}),$$

$$\begin{array}{l} d_{6}=\frac{1}{1152\;c_{0}^{2}d_{2}}(192\;G^{2}H_{0}^{2}d_{2}d_{4}+384\;G^{2}H_{0}d_{0}d_{2}d_{4}+48\;G^{2}H_{0}d_{2}^{3}+192\;G^{2}d_{0}^{2}d_{2}d_{4}+48\;G^{2}d_{0}d_{2}^{3}\\ \;+8\;G^{2}H_{0}d_{4}+8\;G^{2}d_{4}d_{0}+3\;G^{2}d_{2}^{2}-768\;c_{0}^{2}d_{4}^{2}-1536\;c_{0}c_{2}d_{2}d_{4}-384\;c_{0}c_{4}d_{2}^{2}-192\;c_{2}^{2}d_{2}^{2}) \end{array}$$

$$\begin{array}{l} d_{8}=\frac{1}{768\;c_{0}^{2}d_{2}}(144\;G^{2}H_{0}^{2}d_{2}d_{6}+96\;G^{2}H_{0}^{2}d_{4}^{2}+288\;G^{2}H_{0}d_{0}d_{2}d_{6}+192\;G^{2}H_{0}d_{0}d_{4}^{2}\\ \;+112\;G^{2}H_{0}d_{2}^{2}d_{4}+144\;G^{2}d_{0}^{2}d_{2}d_{6}+96\;G^{2}d_{0}^{2}d_{4}^{2}+112\;G^{2}d_{0}d_{2}^{2}d_{4}+6\;G^{2}d_{2}^{4}+3\;G^{2}H_{0}d_{6}\\ \;+3\;G^{2}d_{6}d_{0}+2\;G^{2}d_{4}d_{2}-1152\;c_{0}^{2}d_{4}d_{6}-1152\;c_{0}c_{2}d_{2}d_{6}-768\;c_{0}c_{2}d_{2}^{4}-768\;c_{0}c_{4}d_{2}d_{4}\\ \;-192\;c_{0}c_{6}d_{2}^{2}-384\;c_{2}^{2}d_{2}d_{4}-192\;c_{2}c_{4}d_{2}^{2}) \end{array}$$

$$\begin{array}{l} d_{10}=\frac{1}{28800c_{0}^{2}d_{2}}(5760G^{2}H_{0}^{2}d_{2}d_{8}+8640G^{2}H_{0}^{2}d_{4}d_{6}+11520G^{2}H_{0}d_{0}d_{2}d_{8}+17280G^{2}H_{0}d_{0}d_{4}d_{6}\\ \;+4680G^{2}H_{0}d_{2}^{2}d_{6}+4800G^{2}H_{0}d_{2}d_{4}^{2}+5760G^{2}d_{0}^{2}d_{2}d_{8}+8640G^{2}d_{0}^{2}d_{4}d_{6}+4680G^{2}d_{0}d_{2}^{2}d_{6}\\ \;+4800G^{2}d_{0}d_{2}d_{4}^{2}+960G^{2}d_{2}^{3}d_{4}+72G^{2}H_{0}d_{8}+72G^{2}d_{0}d_{8}+45G^{2}d_{2}d_{6}+20G^{2}d_{4}^{2}-46080c_{0}^{2}d_{4}d_{8}\\ \;-25920c_{0}^{2}d_{6}^{2}-46080c_{0}c_{2}d_{2}d_{8}-69120c_{0}c_{2}d_{4}d_{6}-34560c_{0}c_{4}d_{2}d_{6}-23040c_{0}c_{2}d_{4}^{2}-5760c_{0}c_{8}d_{2}^{2}\\ \;-23040c_{0}c_{6}d_{2}d_{4}-17280c_{2}^{2}d_{2}d_{6}-11520c_{2}^{2}d_{4}^{2}-23040c_{2}c_{4}d_{2}d_{4}-5760c_{2}c_{6}d_{2}^{2}-2880c_{4}^{2}d_{2}^{2}) \end{array}$$

$$\begin{array}{l} d_{12}=\frac{1}{17280c_{0}^{2}d_{2}}(3600G^{2}H_{0}^{2}d_{2}d_{10}+5760G^{2}H_{0}^{2}d_{4}d_{8}+3240G^{2}H_{0}^{2}d_{6}^{2}+7200G^{2}H_{0}d_{0}d_{2}d_{10}\\ \;+11520G^{2}H_{0}d_{0}d_{4}d_{8}+6480G^{2}H_{0}d_{0}d_{6}^{2}+3024G^{2}H_{0}d_{2}^{2}d_{8}+6480G^{2}H_{0}d_{2}d_{4}d_{6}+960G^{2}H_{0}d_{4}^{3}\\ \;+3600G^{2}d_{0}^{2}d_{2}d_{10}+5760G^{2}d_{0}^{2}d_{4}d_{8}+3240G^{2}d_{0}^{2}d_{6}^{2}+3024G^{2}d_{0}d_{2}^{2}d_{8}+6480G^{2}d_{0}d_{2}d_{4}d_{6}\\ \;+960G^{2}d_{0}d_{4}^{3}+630G^{2}d_{2}^{3}d_{6}+880G^{2}d_{2}^{2}d_{4}^{2}+30G^{2}H_{0}d_{10}+30G^{2}d_{0}d_{10}+18G^{2}d_{2}d_{8}+15G^{2}d_{4}d_{6}\\ \;-28800c_{0}^{2}d_{4}d_{10}-34560c_{0}^{2}d_{6}d_{8}-28800c_{0}c_{2}d_{2}d_{10}-46080c_{0}c_{2}d_{4}d_{8}-25920c_{0}c_{2}d_{6}^{2}\\ \;-23040c_{0}c_{4}d_{2}d_{8}-34560c_{0}c_{4}d_{4}d_{6}-17280c_{0}c_{6}d_{2}d_{6}-11520c_{0}c_{6}d_{4}^{2}-11520c_{0}c_{8}d_{2}d_{4}\\ \;-2880c_{0}c_{10}d_{2}^{2}-11520c_{2}^{2}d_{2}d_{8}-17280c_{2}^{2}d_{4}d_{6}-17280c_{2}c_{4}d_{2}d_{6}-11520c_{2}c_{4}d_{4}^{2}\\ \;-11520c_{2}c_{6}d_{2}d_{4}-2880c_{2}c_{8}d_{2}^{2}-5760c_{4}^{2}d_{2}d_{4}-2880c_{4}c_{6}d_{2}^{2}) \end{array}$$

$$\begin{array}{l} d_{14}=\frac{1}{1128960c_{0}^{2}d_{2}}(241920G^{2}H_{0}^{2}d_{2}d_{12}+403200G^{2}H_{0}^{2}d_{4}d_{10}+483840G^{2}H_{0}^{2}d_{6}d_{8}\\ \;+483840G^{2}H_{0}d_{0}d_{2}d_{12}+806400G^{2}H_{0}d_{0}d_{4}d_{10}+967680G^{2}H_{0}d_{0}d_{6}d_{8}+208320G^{2}H_{0}d_{2}^{2}d_{10}\\ \;+462336G^{2}H_{0}d_{2}d_{4}d_{8}+241920G^{2}H_{0}d_{2}d_{6}^{2}+201600G^{2}H_{0}d_{4}^{2}d_{6}+241920G^{2}d_{0}^{2}d_{2}d_{12}\\ \;+403200G^{2}d_{0}^{2}d_{4}d_{10}+483840G^{2}d_{0}^{2}d_{6}d_{8}+208320G^{2}d_{0}d_{2}^{2}d_{10}+462336G^{2}d_{0}d_{2}d_{4}d_{8}\\ \;+241920G^{2}d_{0}d_{2}d_{6}^{2}+201600G^{2}d_{0}d_{4}^{2}d_{6}+44352G^{2}d_{2}^{3}d_{8}+124320G^{2}d_{2}^{2}d_{4}d_{6}+35840G^{2}d_{2}d_{4}^{3}\\ \;+1440G^{2}H_{0}d_{12}+1440G^{2}d_{0}d_{12}+840G^{2}d_{2}d_{10}+672G^{2}d_{4}d_{8}+315G^{2}d_{6}^{2}-1935360c_{0}^{2}d_{4}d_{12}\\ \;-2419200c_{0}^{2}d_{6}d_{10}-1290240c_{0}^{2}d_{8}^{2}-1935360c_{0}c_{2}d_{2}d_{12}-3225600c_{0}c_{2}d_{4}d_{10}-3870720c_{0}c_{2}d_{6}d_{8}\\ \;-1612800c_{0}c_{4}d_{2}d_{10}-2580480c_{0}c_{4}d_{4}d_{8}-1451520c_{0}c_{4}d_{6}^{2}-1290240c_{0}c_{6}d_{2}d_{8}\\ \;-1935360c_{0}c_{6}d_{4}d_{6}-967680c_{0}c_{8}d_{2}d_{6}-645120c_{0}c_{8}d_{4}^{2}-645120c_{0}c_{10}d_{2}d_{4}-161280c_{0}c_{12}d_{2}^{2}\\ \;-806400c_{2}^{2}d_{2}d_{10}-1290240c_{2}^{2}d_{4}d_{8}-725760c_{2}^{2}d_{6}^{2}-1290240c_{2}c_{4}d_{2}d_{8}-1935360c_{2}c_{4}d_{4}d_{6}\\ \;-967680c_{2}c_{6}d_{2}d_{6}-645120c_{2}c_{6}d_{4}^{2}-645120c_{2}c_{8}d_{2}d_{4}-161280c_{2}c_{10}d_{2}^{2}-483840c_{4}^{2}d_{2}d_{6}\\ \;-322560c_{4}^{2}d_{4}^{2}-645120c_{4}c_{6}d_{2}d_{4}-161280c_{4}c_{8}d_{2}^{2}-80640c_{6}^{2}d_{2}^{2}) \end{array}$$

$$\begin{array}{l} d_{16}=\frac{1}{322560c_{0}^{2}d_{2}}(70560\;G^{2}H_{0}^{2}d_{2}d_{14}+120960\;G^{2}H_{0}^{2}d_{4}d_{12}+151200\;G^{2}H_{0}^{2}d_{6}d_{10}+315\;G^{2}H_{0}d_{14}\\ \;+80640\;G^{2}H_{0}^{2}d_{8}^{2}+141120\;G^{2}H_{0}d_{0}d_{2}d_{14}+241920\;G^{2}H_{0}d_{0}d_{4}d_{12}+302400\;G^{2}H_{0}d_{0}d_{6}d_{10}\\ \;+161280\;G^{2}H_{0}d_{0}d_{8}^{2}+61920\;G^{2}H_{0}d_{2}^{2}d_{12}+141120\;G^{2}H_{0}d_{2}d_{4}d_{10}+153216\;G^{2}H_{0}d_{2}d_{6}d_{8}\\ \;+61824\;G^{2}H_{0}d_{4}^{2}d_{8}+60480\;G^{2}H_{0}d_{4}d_{6}^{2}+70560\;G^{2}d_{0}^{2}d_{2}d_{14}+120960\;G^{2}d_{0}^{2}d_{4}d_{12}-40320\;c_{6}c_{8}d_{2}^{2}\\ \;+151200\;G^{2}d_{0}^{2}d_{6}d_{10}+80640\;G^{2}d_{0}^{2}d_{8}^{2}+61920\;G^{2}d_{0}d_{2}^{2}d_{12}+141120\;G^{2}d_{0}d_{2}d_{4}d_{10}+2240\;G^{2}d_{4}^{4}\\ \;+153216\;G^{2}d_{0}d_{2}d_{6}d_{8}+61824\;G^{2}d_{0}d_{4}^{2}d_{8}+60480\;G^{2}d_{0}d_{4}d_{6}^{2}+13440\;G^{2}d_{2}^{3}d_{10}+38304\;G^{2}d_{2}^{2}d_{4}d_{8}\\ \;+19215\;G^{2}d_{2}^{2}d_{6}{}^{2}+31920\;G^{2}d_{2}d_{4}^{2}d_{6}+315\;G^{2}d_{0}d_{14}+180\;G^{2}d_{2}d_{12}+140\;G^{2}d_{4}d_{10}+126\;G^{2}d_{6}d_{8}\\ \;-564480\;c_{0}c_{2}d_{2}d_{14}-967680\;c_{0}c_{2}d_{4}d_{12}-1209600\;c_{0}c_{2}d_{6}d_{10}-645120\;c_{0}c_{2}d_{8}^{2}-483840\;c_{0}c_{4}d_{2}d_{12}\\ \;-806400\;c_{0}c_{4}d_{4}d_{10}-967680\;c_{0}c_{4}d_{6}d_{8}-403200\;c_{0}c_{6}d_{2}d_{10}-645120\;c_{0}c_{6}d_{4}d_{8}-362880\;c_{0}c_{6}d_{6}^{2}\\ \;-322560\;c_{0}c_{8}d_{2}d_{8}-483840\;c_{0}c_{8}d_{4}d_{6}-241920\;c_{0}c_{10}d_{2}d_{6}-161280\;c_{0}c_{10}d_{4}^{2}-161280\;c_{0}c_{12}d_{2}d_{4}\\ \;-40320\;c_{0}c_{14}d_{2}^{2}-241920\;c_{2}^{2}d_{2}d_{12}-40320\;c_{4}c_{10}d_{2}^{2}-403200\;c_{2}^{2}d_{4}d_{10}-483840\;c_{2}^{2}d_{6}d_{8}\\ \;-403200\;c_{2}c_{4}d_{2}d_{10}-645120\;c_{2}c_{4}d_{4}d_{8}-362880\;c_{2}c_{4}d_{6}^{2}-322560\;c_{2}c_{6}d_{2}d_{8}-483840\;c_{2}c_{6}d_{4}d_{6}\\ \;-241920\;c_{2}c_{8}d_{2}d_{6}-161280\;c_{2}c_{8}d_{4}^{2}-161280\;c_{2}c_{10}d_{2}d_{4}-40320\;c_{2}c_{12}d_{2}^{2}-161280\;c_{4}{}^{2}d_{2}d_{8}\\ \;-80640\;c_{6}^{2}d_{2}d_{4}-241920\;c_{4}^{2}d_{4}d_{6}-241920\;c_{4}c_{6}d_{2}d_{6}-161280\;c_{4}c_{6}d_{4}^{2}-161280\;c_{4}c_{8}d_{2}d_{4}) \end{array}$$

$$\begin{array}{l} d_{18}=\frac{1}{3628800c_{0}^{2}d_{2}}(806400\;G^{2}H_{0}^{2}d_{2}d_{16}+1411200\;G^{2}H_{0}^{2}d_{4}d_{14}+1814400\;G^{2}H_{0}^{2}d_{6}d_{12}\\ \;+2016000\;G^{2}H_{0}^{2}d_{8}d_{10}+1612800\;G^{2}H_{0}d_{0}d_{2}d_{16}+2822400\;G^{2}H_{0}d_{0}d_{4}d_{14}\\ \;+3628800\;G^{2}H_{0}d_{0}d_{6}d_{12}+4032000\;G^{2}H_{0}d_{0}d_{8}d_{10}+718200\;d_{2}^{2}G^{2}d_{14}H_{0}\\ \;+1670400\;G^{2}H_{0}d_{2}d_{4}d_{12}+1864800\;G^{2}H_{0}d_{2}d_{6}d_{10}+967680\;G^{2}H_{0}d_{2}d_{8}^{2}\\ \;+739200\;G^{2}H_{0}d_{4}^{2}d_{10}+1451520\;G^{2}H_{0}d_{4}d_{6}d_{8}+226800\;G^{2}H_{0}d_{6}^{3}+806400\;G^{2}d_{0}^{2}d_{2}d_{16}\\ \;+1411200\;G^{2}d_{0}^{2}d_{4}d_{14}+1814400\;G^{2}d_{0}^{2}d_{6}d_{12}+2016000\;G^{2}d_{0}^{2}d_{8}d_{10}+718200\;d_{2}^{2}G^{2}d_{14}d_{0}\\ \;+1670400\;G^{2}d_{0}d_{2}d_{4}d_{12}+1864800\;G^{2}d_{0}d_{2}d_{6}d_{10}+967680\;G^{2}d_{0}d_{2}d_{8}^{2}+739200\;G^{2}d_{0}d_{4}^{2}d_{10}\\ \;+1451520\;G^{2}d_{0}d_{4}d_{6}d_{8}+226800\;G^{2}d_{0}d_{6}^{3}+158400\;G^{2}d_{2}^{3}d_{12}+459200\;G^{2}d_{2}^{2}d_{4}d_{10}\\ \;+468720\;G^{2}d_{2}^{2}d_{6}d_{8}+380800\;G^{2}d_{2}d_{4}^{2}d_{8}+365400\;G^{2}d_{2}d_{4}d_{6}^{2}+100800\;G^{2}d_{4}^{3}d_{6}\\ \;+2800\;G^{2}d_{16}H_{0}+2800\;G^{2}d_{16}d_{0}+1575\;G^{2}d_{2}d_{14}+1200\;G^{2}d_{4}d_{12}+1050\;G^{2}d_{6}d_{10}+504\;G^{2}d_{8}^{2}\\ \;-6451200\;c_{0}c_{2}d_{2}d_{16}-11289600\;c_{0}c_{2}d_{4}d_{14}-14515200\;c_{0}c_{2}d_{6}d_{12}-16128000\;c_{0}c_{2}d_{8}d_{10}\\ \;-5644800\;c_{0}c_{4}d_{2}d_{14}-9676800\;c_{0}c_{4}d_{4}d_{12}-12096000\;c_{0}c_{4}d_{6}d_{10}-6451200\;c_{0}c_{4}d_{8}^{2}\\ \;-4838400\;c_{0}c_{6}d_{2}d_{12}-8064000\;c_{0}c_{6}d_{4}d_{10}-9676800\;c_{0}c_{6}d_{6}d_{8}-4032000\;c_{0}c_{8}d_{2}d_{10}\\ \;-6451200\;c_{0}c_{8}d_{4}d_{8}-3628800\;c_{0}c_{8}d_{6}^{2}-3225600\;c_{0}c_{10}d_{2}d_{8}-4838400\;c_{0}c_{10}d_{4}d_{6}\\ \;-2419200\;c_{0}c_{12}d_{2}d_{6}-1612800\;c_{0}c_{12}d_{4}^{2}-1612800\;d_{2}d_{4}c_{0}c_{14}-403200\;d_{2}^{2}c_{0}c_{16}\\ \;-2822400\;c_{2}^{2}d_{2}d_{14}-4838400\;c_{2}^{2}d_{4}d_{12}-6048000\;c_{2}^{2}d_{6}d_{10}-3225600\;c_{2}^{2}d_{8}^{2}\\ \;-4838400\;c_{2}c_{4}d_{2}d_{12}-8064000\;c_{2}c_{4}d_{4}d_{10}-9676800\;c_{2}c_{4}d_{6}d_{8}-4032000\;c_{2}c_{6}d_{2}d_{10}\\ \;-6451200\;c_{2}c_{6}d_{4}d_{8}-3628800\;c_{2}c_{6}d_{6}^{2}-3225600\;c_{2}c_{8}d_{2}d_{8}-4838400\;c_{2}c_{8}d_{4}d_{6}\\ \;-2419200\;c_{2}c_{10}d_{2}d_{6}-1612800\;c_{2}c_{10}d_{4}^{2}-1612800\;d_{2}d_{4}c_{2}c_{12}-403200\;d_{2}^{2}c_{2}c_{14}\\ \;-2016000\;c_{4}^{2}d_{2}d_{10}-3225600\;c_{4}^{2}d_{4}d_{8}-1814400\;c_{4}^{2}d_{6}^{2}-3225600\;c_{4}c_{6}d_{2}d_{8}\\ \;-4838400\;c_{4}c_{6}d_{4}d_{6}-2419200\;c_{4}c_{8}d_{2}d_{6}-1612800\;c_{4}c_{8}d_{4}^{2}-1612800\;d_{2}d_{4}c_{4}c_{10}\\ \;-403200\;d_{2}^{2}c_{4}c_{12}-1209600\;c_{6}^{2}d_{2}d_{6}-806400\;c_{6}^{2}d_{4}^{2}-1612800\;d_{2}d_{4}c_{6}c_{8}\\ \;-403200\;d_{2}^{2}c_{6}c_{10}-201600\;d_{2}^{2}c_{8}^{2}) \end{array}$$

$$\begin{array}{l} d_{20}=\frac{1}{2016000c_{0}^{2}d_{2}}(453600\;G^{2}H_{0}^{2}d_{2}d_{18}+806400\;G^{2}H_{0}^{2}d_{4}d_{16}+1058400\;G^{2}H_{0}^{2}d_{6}d_{14}\\ \;+1209600\;G^{2}H_{0}^{2}d_{8}d_{12}+630000\;G^{2}H_{0}^{2}d_{10}^{2}+907200\;G^{2}H_{0}d_{0}d_{2}d_{18}+1612800\;G^{2}H_{0}d_{0}d_{4}d_{16}\\ \;+2116800\;G^{2}H_{0}d_{0}d_{6}d_{14}+2419200\;G^{2}H_{0}d_{0}d_{8}d_{12}+1260000\;G^{2}H_{0}d_{0}d_{10}^{2}+408800\;G^{2}H_{0}d_{2}^{2}d_{16}\\ \;+966000\;G^{2}H_{0}d_{2}d_{4}d_{14}+1101600\;G^{2}H_{0}d_{2}d_{6}d_{12}+1176000\;G^{2}H_{0}d_{2}d_{8}d_{10}+432000\;G^{2}H_{0}d_{4}^{2}d_{12}\\ \;+856800\;G^{2}H_{0}d_{4}d_{6}d_{10}+430080\;G^{2}H_{0}d_{4}d_{8}^{2}+393120\;G^{2}H_{0}d_{6}^{2}d_{8}+453600\;G^{2}d_{0}^{2}d_{2}d_{18}\\ \;+806400\;G^{2}d_{0}^{2}d_{4}d_{16}+1058400\;G^{2}d_{0}^{2}d_{6}d_{14}+1209600\;G^{2}d_{0}^{2}d_{8}d_{12}+630000\;G^{2}d_{0}^{2}d_{10}^{2}\\ \;+408800\;G^{2}d_{0}d_{2}^{2}d_{16}+966000\;G^{2}d_{0}d_{2}d_{4}d_{14}+1101600\;G^{2}d_{0}d_{2}d_{6}d_{12}+1176000\;G^{2}d_{0}d_{2}d_{8}d_{10}\\ \;+432000\;G^{2}d_{0}d_{4}^{2}d_{12}+856800\;G^{2}d_{0}d_{4}d_{6}d_{10}+430080\;G^{2}d_{0}d_{4}d_{8}^{2}+393120\;G^{2}d_{0}d_{6}^{2}d_{8}\\ \;+91350\;G^{2}d_{2}^{3}d_{14}+268800\;G^{2}d_{2}^{2}d_{4}d_{12}+279300\;G^{2}d_{2}^{2}d_{6}d_{10}+142128\;G^{2}d_{2}^{2}d_{8}^{2}\\ \;+224000\;G^{2}d_{2}d_{4}^{2}d_{10}+426720\;G^{2}d_{2}d_{4}d_{6}d_{8}+66150\;G^{2}d_{2}d_{6}^{3}+58240\;G^{2}d_{4}^{3}d_{8}+81900\;G^{2}d_{4}^{2}d_{6}^{2}\\ \;+1260\;G^{2}H_{0}d_{18}+1260\;G^{2}d_{18}d_{0}+700\;G^{2}d_{16}d_{2}+525\;G^{2}d_{14}d_{4}+450\;G^{2}d_{12}d_{6}+420\;G^{2}d_{10}d_{8}\\ \;-3628800\;c_{0}c_{2}d_{2}d_{18}-6451200\;c_{0}c_{2}d_{4}d_{16}-8467200\;c_{0}c_{2}d_{6}d_{14}-9676800\;c_{0}c_{2}d_{8}d_{12}\\ \;-5040000\;c_{0}c_{2}d_{10}^{2}-3225600\;c_{0}c_{4}d_{2}d_{16}-5644800\;c_{0}c_{4}d_{4}d_{14}-7257600\;c_{0}c_{4}d_{6}d_{12}\\ \;-8064000\;c_{0}c_{4}d_{8}d_{10}-2822400\;c_{0}c_{6}d_{2}d_{14}-4838400\;c_{0}c_{6}d_{4}d_{12}-6048000\;c_{0}c_{6}d_{6}d_{10}\\ \;-3225600\;c_{0}c_{6}d_{8}^{2}-2419200\;c_{0}c_{8}d_{2}d_{12}-4032000\;c_{0}c_{8}d_{4}d_{10}-4838400\;c_{0}c_{8}d_{6}d_{8}\\ \;-2016000\;c_{0}c_{10}d_{2}d_{10}-3225600\;c_{0}c_{10}d_{4}d_{8}-1814400\;c_{0}c_{10}d_{6}^{2}-1612800\;c_{0}c_{12}d_{2}d_{8}\\ \;-2419200\;c_{0}c_{12}d_{4}d_{6}-1209600\;c_{0}c_{14}d_{2}d_{6}-806400\;c_{0}c_{14}d_{4}^{2}-806400\;c_{0}c_{16}d_{2}d_{4}\\ \;-201600\;c_{0}c_{18}d_{2}^{2}-1612800\;c_{2}^{2}d_{2}d_{16}-2822400\;c_{2}^{2}d_{4}d_{14}-3628800\;c_{2}^{2}d_{6}d_{12}\\ \;-4032000\;c_{2}^{2}d_{8}d_{10}-2822400\;c_{2}c_{4}d_{2}d_{14}-4838400\;c_{2}c_{4}d_{4}d_{12}-6048000\;c_{2}c_{4}d_{6}d_{10}\\ \;-3225600\;c_{2}c_{4}d_{8}^{2}-2419200\;c_{2}c_{6}d_{2}d_{12}-4032000\;c_{2}c_{6}d_{4}d_{10}-4838400\;c_{2}c_{6}d_{6}d_{8}\\ \;-2016000\;c_{2}c_{8}d_{2}d_{10}-3225600\;c_{2}c_{8}d_{4}d_{8}-1814400\;c_{2}c_{8}d_{6}^{2}-1612800\;c_{2}c_{10}d_{2}d_{8}\\ \;-2419200\;c_{2}c_{10}d_{4}d_{6}-1209600\;c_{2}c_{12}d_{2}d_{6}-806400\;c_{2}c_{12}d_{4}^{2}-806400\;c_{2}c_{14}d_{2}d_{4}\\ \;-201600\;c_{2}c_{16}d_{2}^{2}-1209600\;c_{4}^{2}d_{2}d_{12}-2016000\;c_{4}^{2}d_{4}d_{10}-2419200\;c_{4}^{2}d_{6}d_{8}\\ \;-2016000\;c_{4}c_{6}d_{2}d_{10}-3225600\;c_{4}c_{6}d_{4}d_{8}-1814400\;c_{4}c_{6}d_{6}^{2}-1612800\;c_{4}c_{8}d_{2}d_{8}\\ \;-2419200\;c_{4}c_{8}d_{4}d_{6}-1209600\;c_{4}c_{10}d_{2}d_{6}-806400\;c_{4}c_{10}d_{4}^{2}-806400\;c_{4}c_{12}d_{2}d_{4}\\ \;-201600\;c_{4}c_{14}d_{2}^{2}-806400\;c_{6}^{2}d_{2}d_{8}-1209600\;c_{6}^{2}d_{4}d_{6}-1209600\;c_{6}c_{8}d_{2}d_{6}-806400\;c_{6}c_{8}d_{4}^{2}\\ \;-806400\;c_{6}c_{10}d_{2}d_{4}-201600\;c_{6}c_{12}d_{2}^{2}-403200\;c_{8}^{2}d_{2}d_{4}-201600\;c_{8}c_{10}d_{2}^{2}) \end{array}$$

## Appendix C

$$b_{1}=\frac{1}{D-ad_{0}},$$

$$b_{3}=\frac{b_{1}d_{2}}{a(D-ad_{0})},$$

$$b_{5}=\frac{1}{a^{3}(D-ad_{0})}(a^{2}b_{3}d_{2}+b_{1}d_{4}),$$

$$b_{7}=\frac{1}{a^{5}(D-ad_{0})}(a^{4}b_{5}d_{2}+a^{2}b_{3}d_{4}+b_{1}d_{6}),$$

$$b_{9}=\frac{1}{a^{7}(D-ad_{0})}(a^{6}b_{7}d_{2}+a^{4}b_{5}d_{4}+a^{2}b_{3}d_{6}+b_{1}d_{8}),$$

$$b_{11}=\frac{1}{a^{9}(D-ad_{0})}(a^{8}b_{9}d_{2}+a^{6}b_{7}d_{4}+a^{4}b_{5}d_{6}+a^{2}b_{3}d_{8}+b_{1}d_{10}),$$

$$b_{13}=\frac{1}{a^{11}(D-ad_{0})}(a^{10}b_{11}d_{2}+a^{8}b_{9}d_{4}+a^{6}b_{7}d_{6}+a^{4}b_{5}d_{8}+a^{2}b_{3}d_{10}+b_{1}d_{12}),$$

$$b_{15}=\frac{1}{a^{13}(D-ad_{0})}(a^{12}b_{13}d_{2}+a^{10}b_{11}d_{4}+a^{8}b_{9}d_{6}+a^{6}b_{7}d_{8}+a^{4}b_{5}d_{10}+a^{2}b_{3}d_{12}+b_{1}d_{14}),$$

$$b_{17}=\frac{1}{a^{15}(D-ad_{0})}(b_{15}d_{2}a^{14}+b_{13}d_{4}a^{12}+b_{11}d_{6}a^{10}+b_{9}d_{8}a^{8}+b_{7}d_{10}a^{6}+b_{5}d_{12}a^{4}+b_{3}d_{14}a^{2}+b_{1}d_{16}),$$

$$\begin{array}{l} b_{19}=\frac{1}{a^{17}(D-ad_{0})}(b_{17}d_{2}a^{16}+b_{15}d_{4}a^{14}+b_{13}d_{6}a^{12}+b_{11}d_{8}a^{10}+b_{9}d_{10}a^{8}+b_{7}d_{12}a^{6}+b_{5}d_{14}a^{4}\\ \;+b_{3}d_{16}a^{2}+b_{1}d_{18}) \end{array}$$

$$\begin{array}{l} b_{21}=\frac{1}{a^{19}(D-ad_{0})}(b_{19}d_{2}a^{18}+b_{17}d_{4}a^{16}+b_{15}d_{6}a^{14}+b_{13}d_{8}a^{12}+b_{11}d_{10}a^{10}+b_{9}d_{12}a^{8}+b_{7}d_{14}a^{6}\\ \;+b_{5}d_{16}a^{4}+b_{3}d_{18}a^{2}+b_{1}d_{20}) \end{array}$$
